# The antibacterial effect of tellurite is achieved through intracellular acidification and magnesium disruption

**DOI:** 10.1002/mlf2.70028

**Published:** 2025-08-24

**Authors:** Wanli Peng, Yali Fu, Yanqiu Wang, Zixin Deng, Daijie Chen, Shuangjun Lin, Rubing Liang

**Affiliations:** ^1^ State Key Laboratory of Microbial Metabolism, Joint International Research Laboratory of Metabolic & Developmental Sciences, School of Life Sciences and Biotechnology Shanghai Jiao Tong University Shanghai China; ^2^ State Key Laboratory of Microbial Metabolism, School of Pharmaceutical Sciences Shanghai Jiao Tong University Shanghai China; ^3^ Frontiers Science Center for Transformative Molecules Shanghai Jiao Tong University Shanghai China

**Keywords:** antibacterial mechanism, intracellular acidification, magnesium homeostasis, synergistic effect, tellurite

## Abstract

Antibiotic resistance has caused a severe reduction in bacteriostatic action and clinical therapy, demanding effective agents or strategies. Tellurite is an ancient yet powerful antimicrobial agent with an ambiguous mechanism. In this study, we uncovered the underlying action mechanism of tellurite by disturbing the cellular homeostasis of proton and metal ions. Tellurite, entering into *Escherichia coli* MG1655 cells, synchronously imported excess protons and induced intracellular acidification. The intracellular pH declined upon exposure to 0.5 μg/ml of tellurite (the minimal inhibitory concentration, MIC) for 15 min, decreasing from 7.5 to 6.3 in 3 h. A dramatic decrease (31%–73%) in cellular magnesium contents and cytoplastic Mg^2+^ levels occured early after a 5‐min treatment with tellurite, primarily via the enhanced efflux by FocB/MdtL/MdtG and the reduced influx by MgtA/CorA. Disruption of cellular Mg^2+^ homeostasis by tellurite severely hindered ribosome assembly, retarded protein synthesis, and disturbed cellular metabolism. This action logic was applicable to various pathogens. Furthermore, a combination of trace tellurite (0.01/0.1× MIC) synergistically augmented the efficacy of antibiotics at sublethal doses (0.5× MIC) against hypervirulent and drug‐resistant bacterial strains in vitro and in vivo, significantly enhancing the survival rate and the wound‐healing rate of infected animals. These discoveries regarding this metalloid present a promising perspective for combating stubborn and drug‐resistant pathogens.

## Impact statement

Antibiotic resistance poses an escalating threat, making infections more difficult to treat. Our research discloses how tellurite, an age‐old yet potent antimicrobial, disrupts bacterial survival by disturbing the balance of protons and magnesium. This imbalance impedes protein production and weakens bacterial metabolism. Significantly, trace amounts of tellurite markedly enhance the efficacy of existing antibiotics, even against drug‐resistant strains. In the infected animals, this combination improved survival rates and wound healing. These findings present a promising new strategy to combat stubborn bacterial infections and enhance antibiotic treatments, offering hope for tackling antibiotic resistance through a novel, science‐based approach.

## INTRODUCTION

The escalating problem of multidrug resistance has resulted in a considerable reduction/decline in the efficacy of antimicrobial agents, posing a serious threat to global health. Given the prolonged timelines associated with the development and application of new antibiotics, there has been a resurgence of interest in repurposing classic antimicrobial agents^1^. Tellurite (TeO₃²⁻), the oxyanion of the nonessential metalloid tellurium (Te), has exhibited potent antimicrobial activity at strikingly low concentrations (nM–μM)^2^, ^3^. Notably, tellurite has been employed as an effective antimicrobial agent before the emergence of antibiotics, due to its capacity to be metabolized and excreted by the human body^2^, ^3^.

The primary mechanism underlying the antimicrobial action of tellurite has been attributed to the severe depletion of cellular thiols and the generation of reactive oxygen species (ROS)^3^, ^4^. At high concentrations of tellurite, it has been shown that tellurite disrupts the heme biosynthesis in *Escherichia coli,* and the elevated levels of protoporphyrin IX are likely contributing to its toxicity^5^. Nevertheless, this explanation is inadequate in accounting for the high toxicity of tellurite against certain ROS‐resistant bacteria, as well as the relatively low toxicity of selenite, a related compound that induces a more substantial increase in cellular ROS production^5^, ^6^. Furthermore, the impact of accumulated metabolic intermediates remains ambiguous. Despite the fact that the toxicity mechanisms and health effects of tellurite on animals and humans are not fully comprehended, studies have indicated that administration of 2 mM/kg tellurite in mice shows minimal toxicity. Additionally, cytotoxic effects on murine hepatocarcinoma cells are only witnessed at the concentrations as high as 0.1 mM^7^, ^8^. These findings pose the question of whether there exists an alternative mechanism by which tellurite disrupts cellular metabolism and inhibits growth among diverse microorganisms.

It is well documented that the homeostasis of intracellular pH and metal ions is of paramount importance for cell growth and metabolism^9^, ^10^. Virtually all cellular proteins operate optimally within a specific pH range, and the proton motive force (PMF), which is indispensable for energy metabolism, relies heavily on the pH gradient across the cell membrane^9^. Among divalent cations, Mg²⁺ is the most abundant and assumes a crucial role in numerous fundamental processes, such as ribosome stabilization, ATP neutralization, enzyme catalysis, maintenance of membrane potential, and pathogenesis^10^, ^11^, ^12^, ^13^. Certain agents can disrupt the intrabacterial homeostasis of pH or Mg^2+^, resulting in cytotoxic effects and growth inhibition^14^, ^15^. In response to such stresses, bacteria have evolved mechanisms to regulate ion flux and maintain homeostasis^16^. For example, proton‐consuming acid resistance (AR) systems enable bacteria to neutralize excess protons under acid stress.^9^ Likewise, the regulation of cellular Mg^2+^ levels has been demonstrated to enhance the resistance of *Bacillus subtilis* to ribosome‐targeting antibiotics.^17^ Given the rapid and profound influence of tellurite on microorganisms, we hypothesized that its antimicrobial action might be associated with alterations in cation homeostasis through an as‐yet‐unknown mechanism.

In this study, we revealed that tellurite inhibits bacterial growth via an alternative mechanism entailing the disruption of proton and ion homeostasis. Specifically, tellurite elicits intracellular acidification and Mg²⁺ disruption, resulting in ribosome dysfunction and metabolic arrest. This mechanism is widely applicable across various bacterial species. Capitalizing on the universal and effective action of tellurite, we devised a combined therapeutic strategy employing trace amounts of tellurite and sublethal doses of antibiotics to combat multidrug‐resistant pathogens, both in vitro and in vivo. We suggest that tellurite and its analogs have the potential to serve as bacteriostatic or synergistic agents, providing a potential avenue for the development of novel antimicrobial therapies.

## RESULTS

### Tellurite causes a reduction in intracellular pH and magnesium concentration

**Figure 1:**
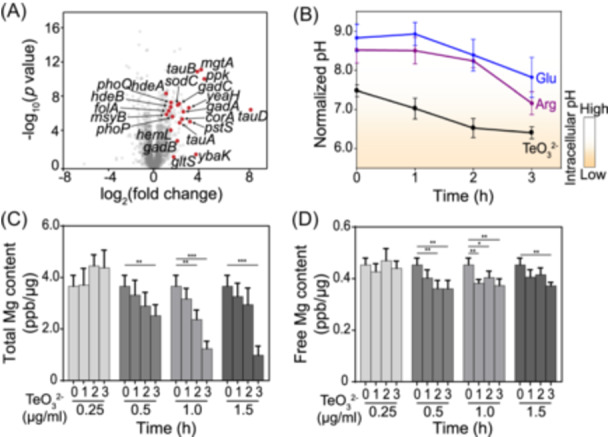
The gene expression, cellular pH, and magnesium content in *Escherichia coli* MG1655 cells are influenced by tellurite. (A) The differential proteomeanalyses of *E. coli* MG1655 under tellurite treatments. A volcano plot of the proteomic data for *E. coli* MG1655 cells treated with 0.5 μg/ml tellurite for 1‒3 h is shown, and the genes with significantly altered expression levels are depicted. (B) The variation of intracellular pH under tellurite treatment. The intracellular pH of *E. coli* MG1655 cells cultivated in media supplemented with tellurite (0.5 μg/ml, pH 7.0), sodium glutamate (40 mM, pH 7.0) or arginine (40 mM, pH 7.0) was determined at 1‒3 h and plotted (*n* = 5; mean ± SD). (C, D) The changes of total magnesium (C) and cytoplasmic Mg^2+^ (D) under tellurite treatments. The contents of total magnesium and cytoplasmic Mg^2+^ in *E. coli* MG1655 cells treated with tellurite (0.25‒1.5 μg/ml) for 1‒3 h were quantified by inductively coupled plasma‐mass spectrometry (ICP‐MS) analysis and plotted after normalization to the total protein concentration (*n* = 5; mean ± SD). *p < 0.05; **p < 0.01; ***p < 0.001.

To assess the cellular responses of *E. coli* MG1655 to tellurite exposure, differential proteomic and transcriptomic analyses were performed. Under tellurite treatment, a significant enrichment of proteins associated with acid stress response (HdeA and HdeB), glutamate‐dependent acid resistance and transport (GadA, GadB, GadC, and GltS), pH/Mg²⁺ homeostasis and regulation (PhoP and PhoQ), Mg²⁺ transport (MgtA and CorA), phosphate homeostasis (Ppk, PstS, and PitA), taurine transport and sulfur metabolism (TauA, TauB, and TauD), as well as flagellar motility (FliG, FlgC, and FlgB) was observed (Figure 1A, Table S1). Notably, in contrast to the cellular responses elicited by Mg²⁺ limitation, tellurite exposure resulted in a more pronounced and rapid induction of ribosome‐associated genes, whereas the genes related to ROS were not significantly upregulated. These transcriptional and proteomic changes became apparent as early as 20 min after exposure to 0.25 μg/ml tellurite, corresponding to half of the minimal inhibitory concentration (MIC) (Figure S1). This finding indicates that tellurite disrupts cellular pH and metal ion homeostasis, along with sulfur and phosphorus metabolism.

Moreover, when *E. coli* MG1655 cells were exposed to 0.5 μg/ml tellurite (MIC), the intracellular pH started to decline within 15 min, dropping from 7.5 to 6.3 over a 3‐h period. A comparable acidification was observed in cells treated with Ag⁺ but not in those exposed to selenite (Figure S3A). Intriguingly, supplementation with 40 mM l‐arginine or l‐glutamate maintained the intracellular pH above 7.5 even after 3 h of tellurite exposure, significantly promoting cell growth (Figures 1B and S2). Given that the cation‐phosphate symporter PitA primarily mediates tellurite import and that glutamate‐ and arginine‐dependent acid resistance (AR) systems confer protection against acid stress^18^, ^19^, these findings suggest that tellurite symports with protons into the cell, resulting in severe intracellular acidification.

Furthermore, in *E. coli* MG1655 cells treated with 0.5 μg/ml tellurite, magnesium efflux was detected as early as 5 min after treatment (Video S1). It resulted in a substantial reduction of more than 31% in total intracellular magnesium content after 3 h treatment (Figure 1C). Exposure to higher concentrations of tellurite (1.0 and 1.5 μg/ml) for 3 h led to even more pronounced decreases, with reductions of 66% and 73%, respectively. Notably, the decline in cytosolic Mg²⁺ levels was observed in the cells treated by tellurite higher than 0.5 μg/ml for 1 h, and 3‐h treatment with tellurite of 1.5 μg/ml further decreased the free Mg²⁺ levels (Figures 1C,D).

Regarding other divalent metal ions, although a notable reduction in cytosolic Mn²⁺ levels was also observed with the accumulation of tellurite in cells, the total intracellular contents of manganese, calcium, and iron remained largely unaltered under all tellurite treatment circumstances (Figure S4). Moreover, the integrity of the cell membrane was sustained throughout the exposure period (Figure S5). Significantly, supplementation with high concentrations of Mg²⁺ (20–100 mM) effectively restored the cell viability suppressed by tellurite (0.25–1.5 μg/ml) in a dose‐dependent manner, and the cells treated by tellurite of 10 μg/ml only recovered by Mg²⁺ of 50 or 100 mM (Figure S6A–F). However, supplementation of Mn²⁺ failed to confer any protective effect, even at an extremely high concentration (500 μM) (Figure S6G–L). These findings indicate that tellurite specifically and profoundly disrupts cellular magnesium homeostasis, resulting in significant Mg²⁺ efflux and reduction, while exerting minimal effects on other essential divalent cations.

### Tellurite induces intracellular acidification before the disruption of Mg^2+^ homeostasis

**Figure 2:**
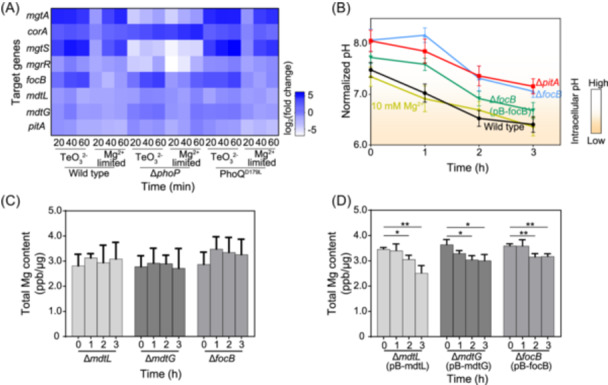
Cellular pH and magnesium content are successively affected by tellurite. (A) The differential transcriptome analyses of several strains under tellurite‐supplied and Mg^2+^‐limited treatments. The transcription levels of six transporter genes and two transport‐associated genes in the wild‐type *Escherichia coli* MG1655, Δ*phoP,* and PhoQ^D179L^ strains cultured in media supplemented with tellurite (0.5 μg/ml) and Mg^2+^‐limited medium were determined at 20‒60 min and normalized to that of the *gapA* gene (*n* = 5; mean ± SD). (B) The changes of intracellular pH in several strains under tellurite treatment and Mg^2+^‐supplied treatments. The intracellular pH of wild‐type *E. coli* MG1655 and three mutant strains (∆*focB*, ∆*focB*(pB*‐focB*), ∆*pitA*) cultured in medium supplemented with tellurite (0.5 μg/ml) and wild‐type cells treated with tellurite and extra Mg^2+^ (10 mM) were determined at 0‒3 h and plotted (*n* = 5; mean ± SD). (C, D) The variation of total magnesium in mutant strains (C) and their complement strains (D) under tellurite treatments. The total magnesium levels in three mutants (∆*mdtL*, ∆*mdtG*, and ∆*focB*) and their complement strains treated with tellurite (0.5 μg/ml) for 0‒3 h were quantified by ICP‒MS analysis and plotted after normalization to total protein levels (*n* = 5; mean ± SD). *p < 0.05; **p < 0.01; ***p < 0.001.

Specific transporters exert a crucial role in modulating fluctuations in cellular pH and Mg²⁺ levels within intact cells. Transcriptional analysis disclosed that tellurite exposure (0.25 and 0.5 μg/ml) promptly induced the expression of *pitA* and seven transporter genes (*rcnA*, *marB*, *alx*, *arsB*, *mdtL*, *mdtG*, and *focB*) within 20 min (Figures 2A and S7, Table S2). This induction was more pronounced and occurred significantly faster than the response elicited by Mg²⁺ limitation (Figure 2A). After a 3‐h tellurite treatment, most knockout mutants exhibited a sharp and substantial reduction in intracellular pH, similar to that observed in wild‐type *E. coli* MG1655 (Figures 2B and S8). Nevertheless, the ∆*pitA* and ∆*focB* strains exhibited only a slight and gradual pH decrease (pH 7.0–7.2) (Figures 2B and S8A). Notably, overexpressing *focB* gene in ∆*focB* strain resulted in a global decrease in intracellular pH, highlighting the crucial role of *focB* gene in pH stabilization under tellurite stress (Figure 2B). Interestingly, despite the beneficial effects of excess Mg²⁺ supplementation on cell growth, it failed to elevate intracellular pH (Figure 2B). These results imply that the fluctuation of cellular pH under tellurite treatment happens independently to the extrusion of magnesium ions and occurs prior to the depletion of intracellular Mg²⁺. The role of these transporters in Mg²⁺ homeostasis was further corroborated by cellular Mg²⁺ measurements in transporter mutants. Under tellurite exposure, total cellular magnesium levels remained relatively stable in the ∆*mdtL*, ∆*mdtG*, and ∆*focB* mutants, in sharp contrast to the pronounced Mg²⁺ disruption observed in wild‐type *E. coli* MG1655 and other transporter mutants (∆*mdtL*, ∆*mdtG*, ∆*focB*, ∆*marB*, ∆*alx*, ∆*arsB*, and ∆*rcnA*) (Figures 2C and S8B). Moreover, the complement of the three transporters (*mdtL, mdtG*, and *focB*) significantly decreased the total cellular Mg²⁺ levels after 2‐h treatment of tellurite, espeicially *focB* and *mdtL*, indicating their involvement in Mg²⁺ efflux under tellurite stress (Figure 2C,D).

In bacteria, the PhoQ/PhoP two‐component system serves as a sensor for acid stress and Mg²⁺ starvation, thereby regulating the transcription of downstream target genes^20^, ^21^. Under both tellurite exposure and Mg²⁺‐limited conditions, the transcriptional analysis disclosed that the transcription of *focB* and *mdtG* genes were upregulated in wild‐type *E. coli* MG1655, as well as in the Δ*phoP* and PhoQᴰ¹⁷⁹ᴸ mutant strains, showing more obvious enhancement in the treatment of tellurite. Similarly, a slight augment in the transcription levels of *mdtL* and *pitA* genes in the two mutants strains under tellurite exposure was also observed. However, the expression of *mgtA*, *mgtS*, and *mgrR* was significantly repressed in the Δ*phoP* strain, while it was conspicuously induced in both wild‐type and PhoQ^D179L^ strains (Figure 2A). These results suggest that the activation of Mg²⁺ transportation mainly relies on the PhoQ/PhoP signaling system. Furthermore, although intracellular acidification and Mg²⁺ reduction induced by tellurite are relatively independent processes, they are also interconnected.

To ascertain whether intracellular acidification directly contributes to Mg²⁺ reduction, bacterial cultures were supplemented with a low concentration of inorganic acid (HCl, pH 3.0) or organic acid salts (sodium benzoate, potassium sorbate, pH 6.0). The results demonstrated that intracellular pH in *E. coli* MG1655 dropped from 7.6 to 6.2 within 1 h of acid treatment, a decline that was both more rapid and pronounced than that observed under tellurite exposure (Figure S9B). Nevertheless, after 2 h of treatment, the degree of intracellular acidification was comparable between the acid‐treated and the tellurite‐exposed cells (Figures 2B and S9B). Additionally, a substantial reduction in cellular Mg²⁺ levels was identified with prolonged acid exposure, particularly in response to HCl treatment (Figures 2B, S9A,B). Furthermore, the transcriptional analysis disclosed that the transcription of *focB*, *mdtL*, *mdtG*, and *pitA* genes was significantly upregulated in response to the stress of organic acids, exerting a stronger inductive effect after 20 min of exposure. However, the expression of genes involved in Mg²⁺ transport, including *mgtA*, *mgtS*, and *mgtR*, was regulated by the PhoQ/PhoP two‐component system and exhibited a notable delay compared to the induction of other transporter genes under the treatment of organic acids (Figure S9C). These findings imply that intracellular acidification can indeed induce Mg²⁺ efflux, thereby uncovering a common mechanistic action between metalloids and organic acid‐based bacteriostatic agents.

### The disruption of Mg^2+^ induced by tellurite inhibits protein synthesis

**Figure 3:**
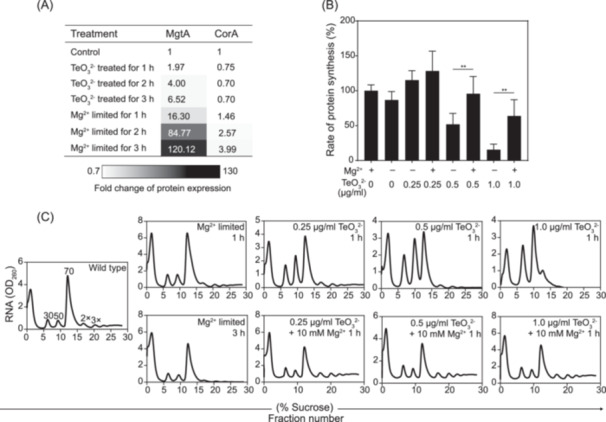
The protein synthesis and ribosome assembly in *Escherichia coli* cells are hindered by tellurite. (A) The expression change of Mg^2+^ transporters under tellurite‐supplied and Mg^2+^‐limited treatments. The expression levels of two Mg^2+^ transporters (MgtA and CorA) in *E. coli* MG1655 cultured in M9 medium with tellurite (0.5 μg/ml) or Mg^2+^‐limited medium were determined at 1‒3 h. The greyscale (bottom) illustrates the fold change in protein expression. (B) The protein synthesis rate analysis of *E. coli* MG1655 under tellurite treatments. Puromycin labeling was conducted on *E. coli* MG1655 cells grown in M9 medium supplemented with tellurite at different concentrations (0.25‒1.0 μg/ml) with/without extra Mg^2+^ (10 mM), and the results were plotted (*n* = 5; mean ± SD). **p < 0.01. (C) The ribosome polysome analyses of *E. coli* MG1655 under different treatments. The ribosome polysome analyses were monitored at 1 and 3 h in cells cultured in M9 medium supplemented with tellurite (0.25‒1.0 μg/ml), M9 medium supplemented with tellurite and extra Mg^2+^ (10 mM), or Mg^2+^‐limited medium. The polysome profiles are representative of five independent experiments.

Mg²⁺ deprivation typically stimulates Mg²⁺ influx to sustain ionic homeostasis and support normal metabolic functions. After 20 min or 1 h of tellurite exposure, the transcriptional analysis disclosed a significant upregulation in the transcription of *mgtA* gene (131.9‐ and 313.0‐fold) and *corA* gene (3.2‐ and 2.8‐fold), which was conspicuously greater than the induction of the two genes observed under Mg²⁺ starvation (*mgtA*, 0.9‐ and 36.9‐fold; *corA*, 0.6‐ and 1.5‐fold) (Figure 2A). Similar upregulated patterns in transcription were also observed in *mgtS* and *mgrR* (Figure 2A). Surprisingly, despite the marked transcriptional activation of Mg²⁺ importers, the cellular Mg²⁺ reduction induced by tellurite could not be compensated. Proteomic analyses revealed that the expression levels of MgtA in wild‐type *E. coli* MG1655 under tellurite treatment were significantly lower than those under Mg²⁺ limitation, with the abundance of MgtA protein reaching merely approximately 1/12 of that in Mg²⁺‐starved conditions (Figure 3A). A similar trend was observed for the expression of MgtS (Figures 3A, S10, and S11). These findings imply a decoupling between transcription and translation, giving rise to a marked delay in protein synthesis. Further exploration via puromycin labeling assays affirmed that tellurite exposure induced a substantial inhibition of protein synthesis in a dose‐dependent manner, whereas excess Mg²⁺ supplementation effectively alleviated this suppression (Figures 3B, S12A,B). Additionally, overexpression of *mgtA* or *corA* augmented the cell survival, especially those under tellurite treatment, while overexpression of the K⁺ transporter *kefB* exerted no detectable effect (Figure S13). Collectively, these outcomes suggest that tellurite‐induced inhibition of protein synthesis aggravates Mg²⁺ reduction, thereby further disrupting cellular Mg²⁺ homeostasis in an irreversible manner.

As Mg²⁺ is indispensable for ribosome assembly, the impaired synthesis of Mg²⁺ transporters disrupts ribosome function^12^. Polysome profiling subsequent to tellurite treatment disclosed that the 30S and 50S ribosomal subunits were unable to assemble effectively into functional 70S ribosomes. When exposed to 0.5 μg/ml tellurite for 3 h or 1.5 μg/ml tellurite for 1 h, ribosomes underwent complete depolymerization, a disruption much more severe than that induced by Mg²⁺ limitation (Figure 3C). Remarkably, this effect was reversed by an excess of Mg²⁺, which facilitated ribosome reassembly (Figures 3C and S12C). While selenite had no impact on ribosome integrity, both Mg²⁺ reduction and ribosome disassembly were observed following Ag⁺ treatment, suggesting a mechanistic resemblance between the antibacterial actions of Ag⁺ and tellurite (Figure S3B–D). Furthermore, tellurite exposure also disrupted *rrs* transcription, the proxy and rate‐limiting step in ribosome biosynthesis. The transcriptional ratio of *rrs* leader RNA to *rrs* RNA significantly increased under tellurite stress but was restored upon supplementation with high concentrations of Mg²⁺ (Figure S14A,B). Additionally, the synthesis and activity of ribosomes are highly energy‐intensive processes that consume a considerable amount of cellular ATP^22^. Even at low tellurite concentrations, a notable increase in intracellular ATP levels was detected, which is detrimental to protein synthesis. This observation suggests that the loss of functional ribosomes further aggravates Mg²⁺ reduction and disrupts normal metabolic processes (Figure S14C,D). In summary, the tellurite‐induced Mg²⁺ disruption results in severe ribosome disassembly, inhibition of protein synthesis, and metabolic disruption^23^.

### Trace amounts of tellurite synergistically interacts with sublethal doses of antibiotics to inhibit bacterial growth

**Figure 4:**
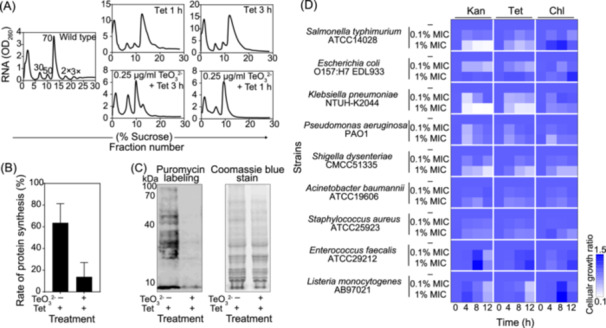
Tellurite exhibits effective synergy effect in combination with traditional antibiotics. (A) The ribosome polysome analyses of *Escherichia coli* MG1655 under tellurite or tetracycline treatments. The ribosome polysome analyses were performed using *E. coli* MG1655 cells treated with tellurite (0.25 μg/ml, 0.5× MIC) and tetracycline (0.3 μg/ml, 0.5× MIC) for 1 and 3 h. The polysome profiles are representative of five independent experiments. (B) The protein synthesis rate analysis of *E. coli* MG1655 under tellurite and tetracycline treatments. Puromycin labeling of the above cultures was performed, and the results were plotted (*n* = 5; mean ± SD). (C) The detection of the protein expression in *E. coli* MG1655 cells treated with tellurite and tetracycline. Western blot analysis (left) and SDS‒PAGE (right) images of the puromycin‐labeling cells in b are shown. The gels are representative of five independent experiments. (D) The growth rate of nine pathogenic strains treated with different combinations of tellurite and antibiotics. The growth inhibition of nine pathogenic strains induced by different combinations of tellurite (0.1% MIC or 1% MIC) and three antibiotics (tetracycline, kanamycin, and chloramphenicol at 0.5× MIC) was measured at four time points (0–12 h) using the broth microdilution checkerboard method. The color bar (right) shows the range of growth ratios compared with that of the cells grown in normal medium (*n* = 5).

Combination therapy constitutes a promising strategy for combating drug‐resistant bacteria^24^. To evaluate whether tellurite displays synergistic effects in conjunction with conventional antibiotics, fractional inhibitory concentration (FIC) index analyses were conducted. The results affirmed that tellurite potentiated the activity of ribosome‐targeting antibiotics, such as tetracycline, kanamycin, and chloramphenicol (Table S3). Co‐treatment with sublethal tellurite (0.5× MIC, 0.25 μg/ml) and tetracycline (0.5× MIC, 0.3 μg/ml) for 1 h significantly compromised ribosome assembly and protein synthesis in *E. coli* MG1655 (Figure 4A–C). In contrast, the combination of tellurite with kanamycin (0.5× MIC, 0.15 μg/ml) or chloramphenicol (0.5× MIC, 0.6 μg/ml) manifested a less remarkable effect after 1 h of treatment. Nevertheless, upon prolonging the treatment duration to 3 h, ribosome disintegration was also witnessed in these combinations (Figure S15). These findings demonstrate that tellurite enhances the efficacy of ribosome‐targeting antibiotics in a synergistic and broad‐spectrum manner, thereby reducing the requisite antibiotic dosage.

Furthermore, to assess the fitness costs associated with the antibacterial mechanism of tellurite, the MICs of tellurite were ascertained for multiple pathogenic bacterial strains, ranging from 0.1 to 500 μg/ml (Table S4). The results demonstrated that exposure to tellurite at MIC levels resulted in a marked reduction of cellular Mg²⁺ and a significant reduction in intracellular pH across nearly all tested pathogenic bacterial strains, to different degrees (Figure S16). The most substantial decreases in total intracellular magnesium content were observed in *Salmonella typhimurium* ATCC 14028, *Klebsiella pneumoniae* HS11286 (a clinical multidrug‐resistant strain), and *Acinetobacter baumannii* ATCC 19606, with Mg²⁺ levels dropping to approximately 15% of the original content (Figure S16A). Similarly, rapid and substantial intracellular acidification was witnessed in *E. coli* EDL933, *K. pneumoniae* NTUH‐K2044, and *Pseudomonas aeruginosa* PAO1, with the most pronounced effect observed in *P. aeruginosa* PAO1 (Figure S16B). These findings revealed that tellurite exerts its antibacterial activity through a conserved mechanism involving intracellular acidification and Mg^2+^ disruption, which is applicable to a broad spectrum of pathogenic bacteria, including hypervirulent and multidrug‐resistant strains.

Furthermore, we assessed the synergistic effects of tellurite in combination with antibiotics on pathogenic bacteria in vitro. Our results indicated that trace amounts of tellurite (0.01 or 0.1× MIC) in conjunction with sublethal concentrations (0.5× MIC) of three antibiotics—tetracycline, kanamycin, and chloramphenicol—effectively hindered the growth of both Gram‐negative and Gram‐positive strains in a dose‐dependent manner (Figure 4D). The most remarkable inhibitory effects were observed for *S. typhimurium*, *K. pneumoniae*, and *Shigella dysenteriae*, where cell growth was suppressed by over 70‐80% (Figure 4D). In summary, tellurite displayed potent synergistic effects with conventional antibiotics in combating pathogenic bacteria. This synergy is mainly mediated by tellurite's capability to disrupt pH and magnesium homeostasis, destabilize ribosomes, and inhibit protein synthesis.

### The combination of tellurite and tetracyclines proves to be effective in combating *K. pneumoniae* infection in vivo

**Figure 5:**
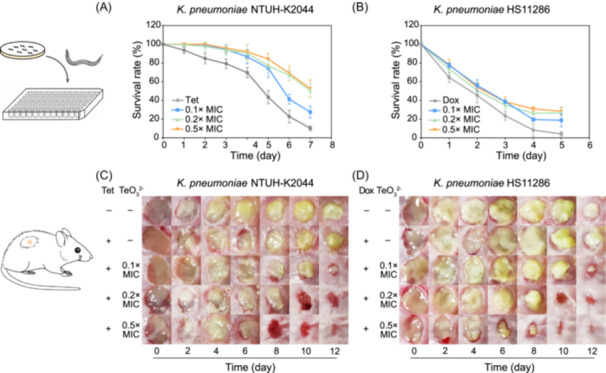
The combination of tellurite and tetracyclines exhibits remarkable antibacterial activity in vivo. (A) The survival rate of *Caenorhabditis elegans* infected with *Klebsiella pneumoniae* NTUH‐K2044 by the combined treatment of tellurite and tetracycline. *C. elegans* infected with *K. pneumoniae* NTUH‐K2044 was treated with tetracycline (5 μg/ml, 2× MIC) solely, or treated with tetracycline and tellurite (0.1‒0.5× MIC); the survival rates over one week were determined and plotted (*n* = 5; mean ± SD). (B) The survival rate of *C. elegans* infected with *K. pneumoniae* HS11286 by the combined treatment of tellurite and doxycycline. *C. elegans* infected with *K. pneumoniae* HS11286 was treated with doxycycline (100 μg/ml, 2× MIC) and with or without tellurite (0.1‒0.5× MIC); the survival rates over 5 days were determined and plotted (*n* = 5; mean ± SD). (C) The wound healing of mice infected with *K. pneumoniae* NTUH‐K2044 by the combined treatment of tellurite and tetracycline. Skin wounds of mice infected with *K. pneumoniae* NTUH‐K2044 were treated with tetracycline (5 μg/ml, 2× MIC) with or without tellurite (0.1‒0.5× MIC). Images of the skin wounds were captured every 2 days for 12 days, and the images are representative of six independent experiments. (D) The wound healing of mice infected with *K. pneumoniae* HS11286 by the combined treatment of tellurite and doxycycline. The skin wounds of mice infected with *K. pneumoniae* HS11286 were treated with doxycycline (100 μg/ml, 2× MIC) with or without tellurite (0.1‒0.5× MIC). Images of the skin wounds were captured every 2 days for 12 days and are representative of six independent experiments.

*K. pneumoniae* is a crucial clinical pathogen associated with high mortality rates and is a prominent member of the “ESKAPE” pathogens, a group that includes *Enterococcus faecium*, *Staphylococcus aureus*, *K. pneumoniae*, *A. baumannii*, *P. aeruginosa*, and *Enterobacter* species. Given the observed synergistic efficacy of tellurite and tetracycline in inhibiting *K. pneumoniae* strains in vitro, we extended our investigation to in vivo models, including a *Caenorhabditis elegans* infection model and a mouse skin infection model. In the *C. elegans* model, worms infected with the hypervirulent *K. pneumoniae* strain NTUH‐K2044 were treated with 5 μg/ml tetracycline (2× MIC) and 0.02–0.1 μg/ml tellurite (0.1–0.5× MIC). Similarly, worms infected with the multidrug‐resistant *K. pneumoniae* strain HS11286 were treated with 200 μg/ml doxycycline (2× MIC) and 0.04–0.2 μg/ml tellurite (0.1–0.5× MIC). The results indicated that the combination of trace tellurite with tetracycline or doxycycline significantly enhanced the survival rate of *C. elegans* compared to antibiotic treatment alone (Figure 5A,B). This synergistic effect was conspicuous on the first day of treatment and persisted throughout the life cycle, culminating in a final rescue ratio of 20%–56% (Figure 5A,B). Analogous synergistic effects were observed when tellurite was combined with kanamycin, chloramphenicol, or streptomycin, leading to a reduction in the required antibiotic dose while improving antibacterial efficacy (Figure S17).

This combination strategy also manifested remarkable therapeutic efficacy in a mouse skin infection model. A round dorsal‐skin incision (6 mm in diameter, 113.0 mm²) was inoculated with *K. pneumoniae* strains NTUH‐K2044 or HS11286. The results indicated that treatment with 0.1–0.5× MIC tellurite in combination with sublethal doses of antibiotics effectively inhibited bacterial growth, decreased the infected area, and expedited wound healing (Figure 5C,D). For infections caused by strain NTUH‐K2044, the wound areas on Day 12 were reduced to 14.5% (13.0 mm²), 5.7% (6.5 mm²), and 4.9% (5.5 mm²) with combination therapy (0.1, 0.2, and 0.5× MIC tellurite, respectively), which were significantly smaller than those in the antibiotic‐only group (69.8%, 78.9 mm²). Similarly, for strain HS11286, the remaining wound areas on Day 12 were 3.5% (3.9 mm²), 1.6% (1.8 mm²), and 0.9% (1.0 mm²), which were substantially smaller than those observed with antibiotic monotherapy (42.7%, 48.3 mm²) (Figure 5C,D). Histological and bacterial analyses further corroborated the efficacy of the combination treatment, revealing increased keratinocyte density, enhanced dermal development, and a significantly lower bacterial load in treated animals (Figure S18). Collectively, these findings demonstrated that the combined application of tellurite and antibiotics offers superior therapeutic outcomes compared to other reported treatments, highlighting its potential as a promising strategy against intractable bacterial infections (Table S5).

## DISCUSSION

The escalating prevalence of drug‐resistant bacteria has notably undermined the efficacy of conventional antibiotic therapies. Monotherapy with antibiotics or single‐modality treatments frequently fails to achieve satisfactory outcomes, particularly when targeting pathogens with a high inclination towards resistance. Combination therapies encompassing bacteriophages or metal nanoparticles have shown potential; nevertheless, their clinical applications are impeded by practical challenges or considerable cytotoxicity^25^, ^26^, ^27^. This has given rise to a refreshed interest in certain long‐established antimicrobial agents.

**Figure 6:**
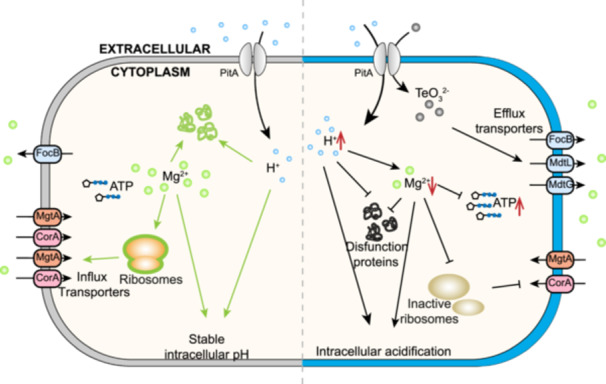
Schematic model of the antibacterial mechanism of tellurite. The cartoon shows that dramatic Mg^2+^ efflux modulation serves as a prompt and effective cellular response to cope with the severe intracellular acidification caused by the symport of tellurite and protons, resulting in ribosome dysfunction, the blockade of protein synthesis, and metabolic interference (left: normal cell, left; right: tellurite‐treated cell).

Tellurite, a historically recognized and widely employed antibacterial compound, has been demonstrated to effectively inhibit biofilm‐forming bacteria and persister cells^28^, ^29^. Nevertheless, its exact mechanism of action remains undetermined, although the depletion of cellular thiols and the accumulation of ROS have been suggested as the main contributors^4^, ^5^, ^30^. In this study, we proposed a hitherto uncharacterized mechanism through which tellurite inhibits bacterial growth, focusing on its disruption of proton and magnesium homeostasis—a mechanism that is widely applicable across diverse bacterial species. Once entering the cells, tellurite promptly induces acidification, which subsequently disrupts Mg²⁺ homeostasis, ultimately leading to the inhibition of protein synthesis, metabolic disruption, and growth arrest. This alternative mode of action establishes a direct connection between the antibacterial effects of nonessential metalloids and the intracellular homeostasis of essential metal ions, a principle that extends to acid‐based preservatives and heavy metals (Figure 6). Furthermore, by taking advantage of the synergistic interaction between tellurite and ribosome‐targeting antibiotics, we developed a combination therapy that effectively rescues pathogen‐infected animal models using sublethal concentrations of tellurite and reduced antibiotic dosages. This approach enhances therapeutic efficacy while reducing antibiotic burden, offering a promising strategy for combating drug‐resistant bacterial infections.

Intracellular pH homeostasis is indispensable for cellular metabolism, as fluctuations in pH can profoundly affect bacterial growth and survival^9^, ^10^. The glutamic acid‐dependent acid resistance (GDAR) system assumes a crucial role in maintaining pH stability in bacteria^9^, ^31^. In *E. coli* cells exposed to tellurite, the prompt and vigorous activation of GadA/GadB, GadC, and GltS facilitates proton disposal subsequent to tellurite entry, while HdeA and HdeB bind to and stabilize acid‐denatured proteins^32^. Concurrently, bacterial cells actively extrude metal ions to preserve cationic homeostasis. The formate–nitrite transporter FocB has been implicated in cellular formate export^33^. Intriguingly, it is also upregulated in response to excess intracellular protons and contributes to the export of Mg²⁺, thereby stabilizing membrane potential and the PMF, both of which are disrupted by tellurite import. This response further aggravates intracellular Mg²⁺ disruption. Additionally, two transporters from the major facilitator superfamily, MdtL and MdtG, might perform analogous functions^34^. Moreover, four metal transporters—RcnA, MarB, Alx, and ArsB—seem to be implicated in the bacterial stress response to tellurite, assisting in the maintenance of metal ion equilibrium between the intracellular and extracellular environments^35^, ^36^.

Metal ions exert a crucial function in bacterial metabolism and are subjected to strict regulatory control^10^. Bacterial cells maintain intracellular concentrations of Mg²⁺, Mn²⁺, and Ca²⁺ at approximately 20 mM, 5 μM, and 0.1 μM, respectively, with Mg²⁺ being indispensable for numerous essential cellular processes^17^, ^37^, ^38^, ^39^. The PhoQ/PhoP two‐component system senses Mg²⁺ and facilitates Mg²⁺ uptake via MgtA and CorA, while additional regulatory mechanisms, including the release of bound Mg²⁺ and modulation through MgtS and mgrR, assist in maintaining cytoplasmic Mg²⁺ homeostasis^40^, ^41^, ^42^.

Our findings revealed that tellurite induces rapid intracellular acidification and Mg²⁺ efflux, with significant alterations taking place within 15 min of exposure. This accounts for the notable upregulation of phoQ/phoP and Mg²⁺ transporter genes observed after 20 min of treatment. The considerable reduction in total cellular magnesium following tellurite exposure is employed to compensate for the sudden loss of cytosolic Mg²⁺. Nevertheless, the disruption of the translation machinery compromises the efficiency of Mg²⁺ influx, thereby impeding metabolic recovery. Consequently, tellurite exerts a continuous and multi‐faceted influence on Mg²⁺ homeostasis.

Furthermore, the complex interplay between magnesium and phosphate homeostasis adds an additional layer of complexity^43^. The phosphate and tellurite transporter PitA also functions as an Mg²⁺ export channel; its transcription and activity are repressed by *mgrR* and MgtS under Mg²⁺ limitation, concurrently with the induction of the phosphate regulon PhoRB^18^, ^44^. The effect of tellurite on phosphate homeostasis is further manifested in the altered expression of PpK and PstS, highlighting its broader influence on macromolecular biosynthesis and energy metabolism.

Cellular processes are highly contingent on intracellular homeostasis, a principle that is applicable not only to prokaryotic cells but also to eukaryotic systems, where disruptions in ionic balance have been associated with the advancement of cancer and cardiovascular diseases^45^, ^46^. Although the effective concentration of tellurite necessary to affect eukaryotic cells is over 100‐fold higher than that for prokaryotic cells, exposure to tellurite at its MIC induces substantial intracellular acidification and Mg²⁺ reduction in HeLa cells (50 μg/ml) and MCF7 cells (100 μg/ml) (Figure S19). These discoveries imply that tellurite might target similar metabolic pathways in both prokaryotic and eukaryotic cells, a hypothesis further substantiated by its inhibitory effects on mitochondrial ribosomal proteins^47^.

At high concentrations, tellurite is known to induce the production of ROS production, resulting in the depletion of cellular thiols^3^. Nevertheless, our findings suggest a minimal upregulation of the ROS‐associated genes in response to low‐concentration tellurite exposure. The modest reduction in intracellular manganese levels and the inability of Mn²⁺ to restore bacterial growth imply that the accumulation of ROS is likely a secondary consequence of Mg²⁺ imbalance. This is further substantiated by the upregulation of the TauABCD system, which alleviates tellurite's competitive interference with sulfite metabolism^48^. This observation also provides a plausible account for the relatively low toxicity of selenite to microorganisms and the enhanced toxicity of tellurite to ROS‐resistant bacteria. Additionally, our previous study discloses that tellurite disrupts heme biosynthesis^5^. Given that key enzymes in the heme biosynthetic pathway, such as HemL, HemB, and HemF, are highly dependent on Mg²⁺ and pH stability^49^, ^50^, we conclude that tellurite‐induced cytoplasmic acidification and Mg²⁺ disruption directly and profoundly compromise cellular respiration. This elucidates the marked toxicity of tellurite to bacteria under anaerobic conditions. Furthermore, our findings demonstrate that commonly used low‐molecular‐weight organic acid salts, such as sodium benzoate and potassium sorbate, inhibit bacterial growth in a manner similar to tellurite, while inorganic acids display slightly different mechanisms. Notably, the antibacterial mechanism of tellurite is also applicable to heavy metal‐based bacteriostatic agents, such as Ag⁺, providing a mechanistic foundation for their effective combined utilization in pathogen eradication^51^.

Elucidating the mechanism of tellurite action lays a foundation for its application as both a bacteriostatic agent and an antibiotic synergist. Trace amounts of tellurite have been shown to enhance the efficacy of multiple antibiotics against ESKAPE pathogens both in vitro and in vivo, with particularly pronounced synergy observed with ribosome‐targeting antibiotics. Tellurite‐induced intracellular acidification and Mg²⁺ disruption facilitate the accumulation and activity of tetracycline, and also enhance the ability of chloramphenicol to compromise membrane integrity^52^. The modulation of ion flux by tellurite thus represents the mechanistic basis for its capacity to significantly enhance pathogen inhibition while reducing the required antibiotic dosage. Furthermore, the PhoQ/PhoP system can be activated by various cationic antimicrobial peptides, such as polymyxin B, which serves as a last‐line therapeutic agent against multidrug‐resistant bacteria^53^. Tellurite displayed a slight synergistic effect with polymyxin B against *E. coli* MG1655, as indicated by a FIC index of 0.87 (Table S3). This synergy might arise from the reductions in outer membrane integrity or the disruptions in membrane potential induced by polymyxin B, which could facilitate intracellular Mg²⁺ efflux^54^. Nevertheless, no synergistic effect was observed against *S. aureus* ATCC 25923, with the FIC index of 1.67 (Table S3), probably due to the restricted bactericidal activity of polymyxin B against Gram‐positive bacteria^54^.

Under various stress circumstances, bacterial cells frequently adopt the formation of biofilms and decelerate their growth as a survival tactic, facilitating resistance to adverse environments and the potential for metabolic recuperation when conditions ameliorate. This adaptive response is particularly prevalent in pathogenic strains^55^. Conventional antibiotics are often proved ineffective at eradicating bacterial cells situated at the core of biofilms or those that have transitioned into a persister state^55^. Although certain small molecules, such as D‐amino acids and the polyamine norspermidine, can partially inhibit biofilm formation, their practical applications remain constrained^56^, ^57^. Tellurite has been proposed to promote biofilm formation via c‐di‐GMP signaling^58^, yet it has also been reported to enhance the efficacy of antibiotics and silver nitrate against biofilms and persister cells^51^. In this study, we discovered that low concentrations of tellurite did not stimulate biofilm formation in nearly all tested bacterial strains. However, at concentrations significantly surpassing the MIC, tellurite conspicuously enhanced biofilm formation in *K. pneumoniae* NTUH‐K2044 and *Salmonella enterica* ATCC 14028, despite the undetectable cell growth (Figure S20). This might represent a bacterial stress response mechanism. In contrast, high concentrations of tellurite inhibited biofilm formation in Gram‐positive bacteria after 48 h, even at concentrations far below their MIC (Figure S20). This notable difference could be ascribed to the distinct biofilm‐forming mechanisms utilized by Gram‐positive and Gram‐negative bacteria^59^.

*K. pneumoniae* is acknowledged as a crucial threat in clinical treatment on account of its high virulence and multidrug resistance. This pathogen is frequently implicated in pneumonia and subcutaneous infections, frequently resulting in necrotizing skin and soft tissue infections^60^, ^61^. In this study, the combined approach of trace tellurite and sublethal doses of antibiotics conspicuously improved both the survival rate and wound healing rate in animal models infected with hypervirulent and multidrug‐resistant *K. pneumoniae* strains. Given that the effective concentrations of tellurite are within safe limits for both animals and humans, tellurite and its derivatives possess considerable potential as antimicrobial agents, functioning either as independent bacteriostatic compounds or as universal adjuvants in antimicrobial therapy^62^.

However, it is of paramount importance to note that tellurite resistance genes are prevalently distributed among various organisms, encompassing microorganisms and plants, and that high tolerance to tellurite is frequently associated with augmented pathogenicity in clinical isolates^63^, ^64^, ^65^. This implies that tellurite resistance genes play a pivotal role in counteracting environmental stresses, including fluctuations in proton and metal ion homeostasis. Nevertheless, our findings manifest that the combination of tellurite with antibiotics remains effective in inhibiting the growth of bacterial strains possessing tellurite resistance genes. Beyond their well‐known functions in oxidation–reduction reactions, recent studies indicate that tellurite resistance genes might enhance microbial adaptability within the gut microbiota and could also be involved in metal ion transport^52^, ^53^. These insights might offer a novel approach for the development of targeted therapies or new strategies to combat multidrug‐resistant pathogens.

## MATERIALS AND METHODS

### Strains, cultures, and chemicals

The strains and plasmids used in this study are listed in Tables S6, S7, and S8. All the knockout mutant strains were constructed with the CRISPR‐Cas9 system and verified by DNA sequencing^66^. Bacterial cells were grown in the modified M9 medium (1× M9 salts, 2 mM MgSO_4_, 0.1 mM CaCl_2_, 4 g/l glucose, 2 g/l amicase, pH 7.0) at 37°C with shaking, unless otherwise indicated. Mueller–Hinton broth (MHB) was purchased from Hope Biotechnology. Eukaryotic cells were cultured with Dulbecco's Modified Eagle Medium (DMEM) containing 10% fetal bovine serum (FBS) and penicillin‐streptomycin, at 37°C, 5% CO_2_. DMEM was obtained from Sangon Biotech.

### Inductively coupled plasma–mass spectrometry (ICP‐MS) analysis

The *E. coli* cells were cultured, collected, and washed with PBS three times before being transferred to precleaned centrifuge tubes (ANPEL Laboratory Technologies, Inc.). After undergoing digestion with 2 ml of ultrapure HNO_3_ at 125°C for 2 h, the samples were diluted with deionized water to 50 ml. The amounts of total protein were normalized, and the samples were analyzed on a Perkin Elmer NexION2000 (PerkinElmer, U.S. LLC.).

### General protein analysis

The membrane proteins of *E. coli* cells were extracted as delineated previously^67^. The cells were harvested and re‐suspended in buffer A (20 mM Tris, 150 mM NaCl, pH 8.0) for sonication. The cell debris was centrifuged at 4°C and 18,000 rpm for 1 h to segregate the cell membranes. After solubilization with buffer B (20 mM Tris, 300 mM NaCl, 1% *N*‐dodecyl‐β‐d‐maltoside, pH 8.0), the membrane proteins were separated by centrifugation at 4°C and 18,000 rpm for 1 h. Subsequently, SDS‒PAGE (sodium dodecyl sulfate‒polyacrylamide gel electrophoresis) and western blot analysis analyses were conducted. The monoclonal antibody against RpoB was purchased from Biolegend; the monoclonal antibody against FLAG and the secondary anti‐mouse antibodies were obtained from Proteintech Group, Inc.

### Differential proteomic analyses

The cells were cultured and harvested to concentrate the total proteins. Subsequently, after being treated with 1 M dithiothreitol (DTT) and 1 M iodoacetamide (IAA) and digested with trypsin (Promega), the peptide mixture was obtained and dissolved in 0.1% formic acid. Peptide analysis was conducted with an Easy nLC 1200‐Q Exactive plus system (Thermo Fisher Scientific) on a C18 column (75 × 250 mm, 3 μm) and eluted at a flow rate of 300 nl/min for 120 min. The spectra were acquired in ESI+ mode with a source temperature of 275°C and a spray voltage of 1.8–2.4 kV. All the data were processed with Protein Discoverer software V2.5 (Thermo Fisher Scientific) using the National Center for Biotechnology Information (NCBI) database.

### Protein synthesis rate and polysome analyses

Protein synthesis was monitored using a nonradioactive method as previously described^68^. The cells were sampled and labeled with 10 μg/ml puromycin at 37°C for 30 min. The incorporation of puromycin was detected through immunoblotting with a monoclonal antibody against puromycin (BioLegend). The cells were collected for polysome analysis and lysed to separate polysomes by sucrose density gradient centrifugation^69^. The sucrose concentrations were analyzed using a Gradient Profiling system (BioComp).

### Determination of minimum inhibitory concentration (MIC)

The determination of bacteria MIC was performed as before described^70^. Briefly, the overnight bacterial cultures originating from single colonies were diluted 1:100 in 200 μl of fresh Mueller‐Hinton Broth (MHB) medium and incubated at 37°C for 16–20 h. The MIC was defined as the lowest concentration of antimicrobial agent that completely inhibited visible growth of the tested isolate.

For eukaryotic cells, the sensitivity to tellurite was determined by the Cell Counting Kit‐8 (CCK‐8) assay (Beyotime Biotechnology). Cells were seeded at a density of 1 × 10^4^ cells/well in 100 μl of Dulbecco's Modified Eagle Medium (DMEM) in 96‐well plates and cultured for 24 h at 37°C. Subsequently, cells were exposed to various concentrations of tellurite for 24–72 h, and then added with the solution of CCK‐8 (10 μl). After extra 2‐h incubation, the absorbance at 450 nm (A450) was measured using a microplate spectrophotometer.

### Determination of biofilm formation

The determination of bacteria biofilm formation was conducted as previously described^71^. Briefly, overnight bacterial cultures were diluted in fresh MHB medium, transferred to 96‐well microtiter plates, and incubated at 37°C for 3 days. After incubation, the liquid cultures were removed, and the wells were washed three times with PBS. Subsequently, the biofilms were fixed with methanol for 15 min, stained with 0.1% crystal violet for 5 min, and washed with PBS. The stained biofilms were resuspended in 33% acetic acid solution, and absorbance was measured at 595 nm.

### RNA extraction and quantitative PCR

Total RNA was extracted using Total RNA Extraction Reagents (TaKaRa), and cDNA was synthesized using a PrimeScript Reverse Transcriptase Kit (TaKaRa). Quantitative PCR was performed with Premix ExTaq (TaKaRa) in a QuantStudio 3 Real‐Time PCR system (Thermo Fisher Scientific), and the *gapA* gene was used as a reference.

### Intracellular pH and ATP measurement

The intracellular pH of bacteria was determined using the plasmid pBAD18‐pHluorin‐mCherry as described previously^72^. Briefly, l‐arabinose was used to initiate the expression of fluorescent proteins. The intracellular pH was calculated based on the intensity ratio of pHluorin/mCherry with a standard curve. The fluorescence of pHluorin (excitation/emission, 479 nm/520 nm) and mCherry (excitation/emission, 579 nm/616 nm) was measured using a Synergy H1 Reader (BioTek Instruments, Inc.). The cellular ATP levels were quantified with a BacTiter‐Glo microbial cell viability assay kit (Promega Corporation) in a Synergy H1 Reader (BioTek Instruments, Inc.) in accordance with the manufacturer's instructions.

For eukaryotic cells, the cellular pH and intracellular Mg^2+^ were respectively determined with BCECF‐AM (excitation/emission, 488 nm/535 nm) (Beyotime Biotechnology) and Mag‐Fura‐2, AM (excitation/emission, 488 nm/517 nm) (Thermo Fisher Scientific). The BCECF‐AM and Mag‐Fura‐2 AM were purchased from Beyotime Biotechnology and Thermo Fisher Scientific, respectively.

### Construction of an in vivo *C. elegans* infection model

The mouse experiments were performed in Henan Sinogene Biotechnology Co., Ltd., including the construction of defective skin mouse and the in vivo infection experiments. The in vivo infection of *C. elegans* was performed as previously described^73^. The *C. elegans* AU37 [*sek‐1* (*km4*); *glp‐4* (*bn2*)] was grown at 20°C on nematode growth media (NGM) plates and seeded with *E. coli* OP50 as a food source. After 12 h of culture, the worms were transferred to the plates and then cultured at 15°C for laying eggs and at 25°C for sterility. The worms were synchronized twice with sterile young adults. The adult worms were harvested, transferred to NGM agar plates, and seeded with a lawn of *K. pneumoniae* NTUH‐K2044 or HS11286 for infection. After 36‐h infection, the worms were harvested, washed, transferred to 96‐well microplates, and incubated with antibiotics and/or tellurite at various concentrations at 25°C. At least 200 worms were used for observation in each group, and the survival rate was calculated daily.

### Construction of an in vivo mouse wound model

The in vivo mouse defective skin infection experiment was conducted as previously described^74^. BALB/c female mice (6–8 weeks) were intraperitoneally injected with 1% pentobarbital sodium (50 mg/kg) for general anesthesia. The back centers of the mice were shaved and disinfected, and a 6 mm‐diameter circle of whole skin was removed to create an acute skin wound. The strain NTUH‐K2044 (1 × 10^8^ CFU) or HS11286 (1 × 10^7^ CFU) was inoculated on the wounds, fully absorbed, and then covered with a sterilized dressing for 48 h. The wound healing process was evaluated by the wound area based on the migrating epithelia and photographed. The whole scab on the wound was removed and homogenized, and the lysate was plated on LB agar for the bacterial survival assay. Bacterial growth on wounds was determined by plate spreading.

### Histological examination

The wound tissue and surrounding skin were removed from the wound bed and fixed immediately with 4% paraformaldehyde (PFA) fixative solution. The fixed tissues were prepared for paraffin sectioning and histopathological investigation. Histological analysis was performed with hematoxylin and eosin, Masson's trichrome, and Giemsa staining.

### Scanning electron microscopy (SEM) and transmission electron microscopy (TEM) imaging

The bacterial cells were collected, washed with PBS twice, and fixed with 2.5% glutaraldehyde. For TEM, the cells were fixed with 2% osmium tetroxide, dehydrated with graded ethanol, and infiltrated with resin for polymerization. The sections of fixed samples were collected and visualized with a Tecnai G2 Spirit Biotwin system (Thermo Fisher Scientific). For SEM, the bacterial cells were dried with a Leica EM CPD300 Critical Point Dryer (Leica Microsystems, GER) and visualized using a TESCAN MAGNA system (TESCAN Group, CR).

### Statistics and reproducibility

Three to five independent experiments were conducted, with three to five replicates for each sample. All the data are presented as the mean value along with the standard deviation. Statistical analysis was conducted using Student's unpaired *t‐*test. A *p* value less than 0.05 was regarded as indicating statistical significance (*p < 0.05, **p < 0.01, ***p < 0.001).

## AUTHOR CONTRIBUTIONS

**Wanli Peng**: Data curation; formal analysis; investigation; methodology; validation; visualization; writing—original draft. **Yali Fu**: Data curation; investigation; methodology. **Yanqiu Wang**: Data curation; investigation; methodology. **zixin deng**: Resources; supervision. **Daijie Chen**: Methodology; resources. **Shuangjun Lin**: Formal analysis; project administration; resources; software; supervision; writing—review and editing. **Rubing Liang**: Conceptualization; data curation; formal analysis; funding acquisition; methodology; project administration; resources; software; supervision; validation; writing—review and editing.

## ETHICS STATEMENT

This study was approved by the Animal Ethics Committee of Henan Sinogene Biotechnology Co., Ltd. (IACUC Issue No. ZXNG‐2023082901).

## CONFLICT OF INTERESTS

The authors declare no conflicts of interests.

## Supporting information

Fig‐S1.

Fig‐S2.

Fig‐S3.

Fig‐S4.

Fig‐S5.

Fig‐S6.

Fig‐S7.

Fig‐S8.

Fig‐S9.

Fig‐S10.

Fig‐S11.

Fig‐S12.

Fig‐S13.

Fig‐S14.

Fig‐S15.

Fig‐S16.

Fig‐S17.

Fig‐S18.

Fig‐S19.

Fig‐S20.

20250318‐Supplementary material.

Video 1.

## Data Availability

All data needed to evaluate the conclusions in the paper are present in the paper and/or the Supplementary Materials.
