# Supplementary material for: The antibacterial effect of tellurite is achieved through intracellular acidification and magnesium disruption
Source: mLife. 2025 Aug 24;4(4):423–36. doi: 10.1002/mlf2.70028 (PMC12395589; doi:10.1002/mlf2.70028)
Supplement: Supplementary file 1 — Fig‐S1. [file MLF2-4-423-s022.pdf]

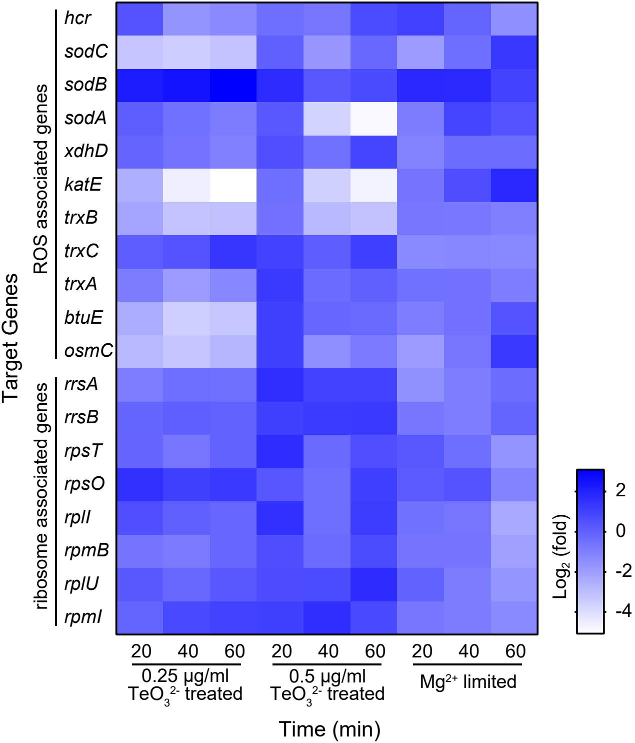
Target Genes
ROS associated genes
ribosome associated genes
hcr
sodC
sodB
sodA
xdhD
katE
trxB
trxC
trxA
btuE
osmC
rrsA
rrsB
rpsT
rpsO
rplI
rpmB
rplU
rpmI
20 40 60
0.25 μg/ml TeO3 2- treated
20 40 60
0.5 μg/ml TeO3 2- treated
20 40 60
Mg2+ limited
Time (min)
Log2 (fold)
2
0
-2
-4
