# Supplementary figures and images for: The antibacterial effect of tellurite is achieved through intracellular acidification and magnesium disruption
Source: mLife. 2025 Aug 24;4(4):423–36. doi: 10.1002/mlf2.70028 (PMC12395589; doi:10.1002/mlf2.70028)

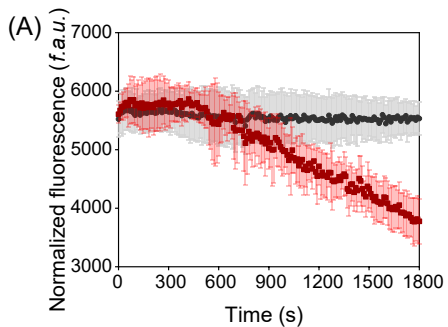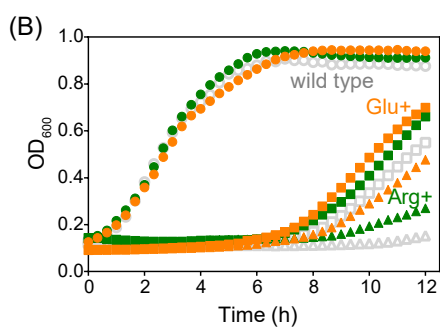

Supplement: Supplementary file 2 — Fig‐S2. [file MLF2-4-423-s004.pdf]

(A)

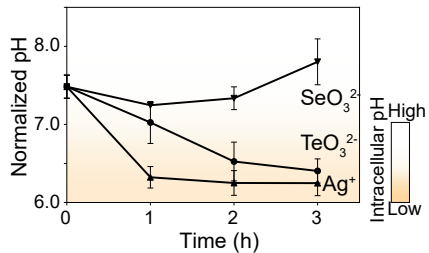

(B)

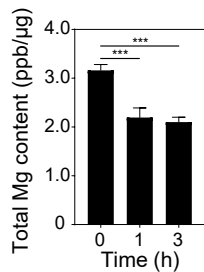

(C)

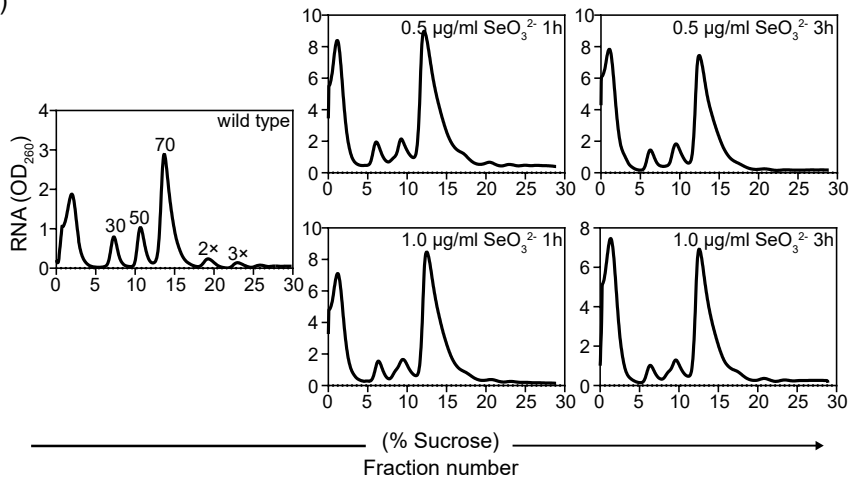

(D)

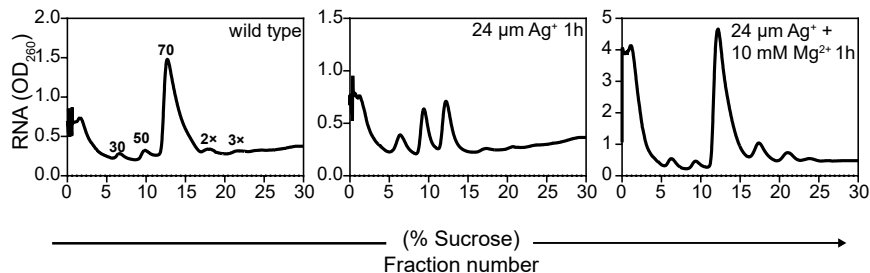

Supplement: Supplementary file 3 — Fig‐S3. [file MLF2-4-423-s010.pdf]

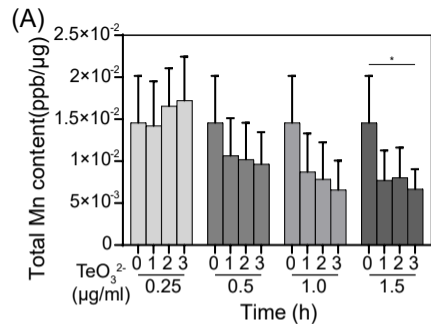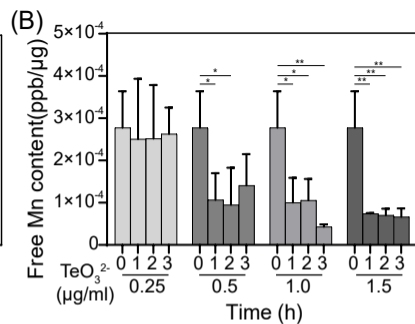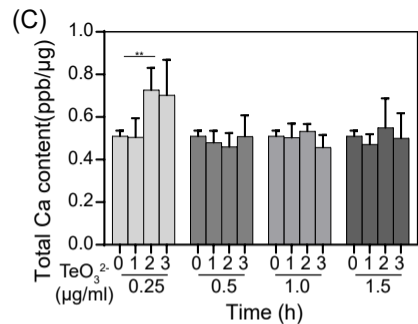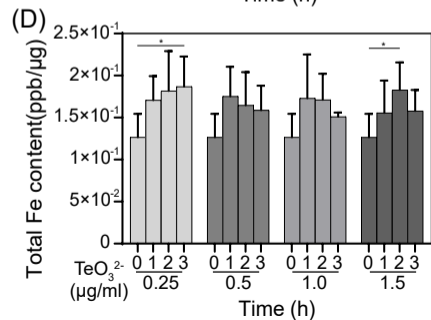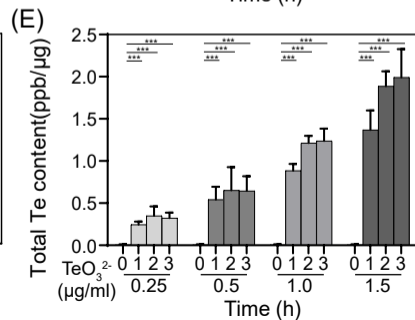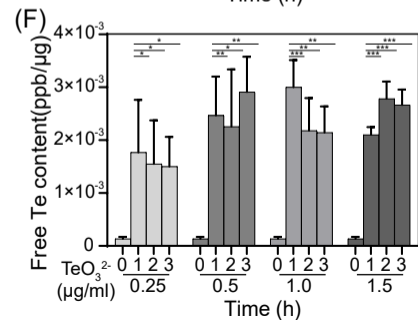

Supplement: Supplementary file 4 — Fig‐S4. [file MLF2-4-423-s006.pdf]

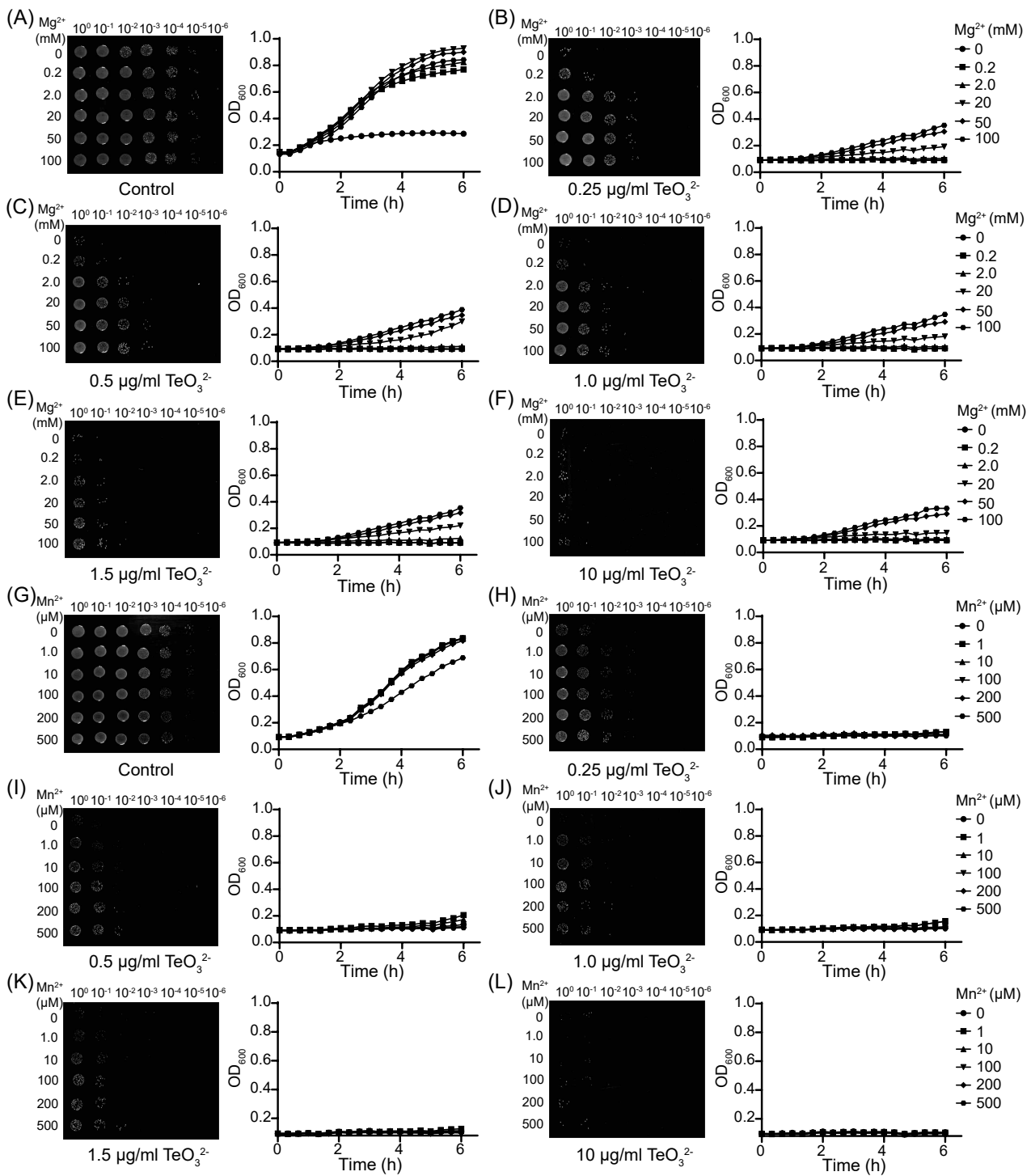

Supplement: Supplementary file 6 — Fig‐S6. [file MLF2-4-423-s014.pdf]

Target Genes

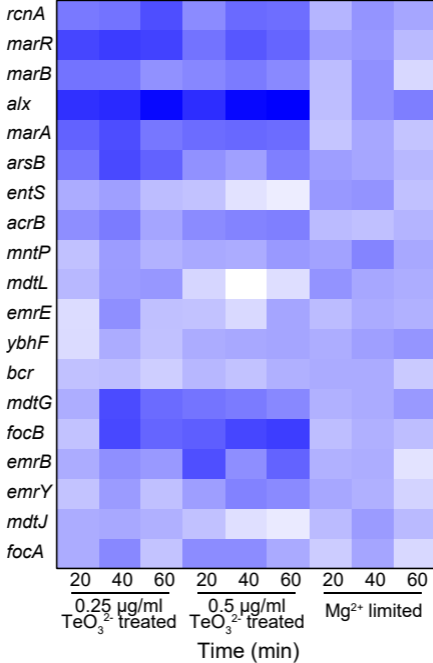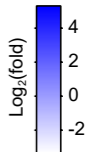

Supplement: Supplementary file 7 — Fig‐S7. [file MLF2-4-423-s009.pdf]

(A)

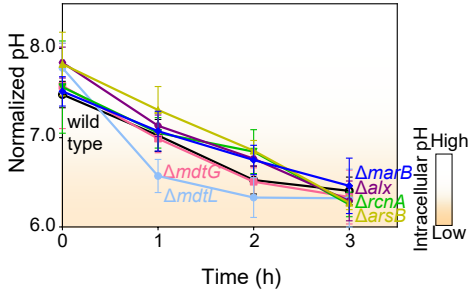

(B)

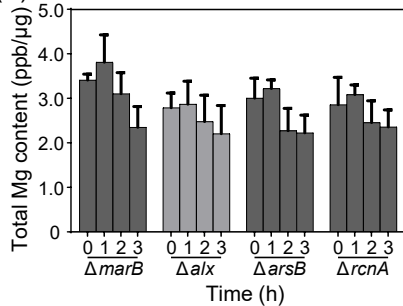

Supplement: Supplementary file 8 — Fig‐S8. [file MLF2-4-423-s011.pdf]

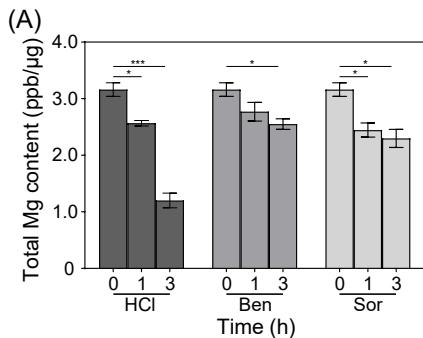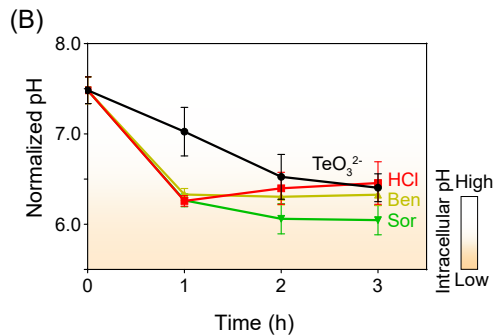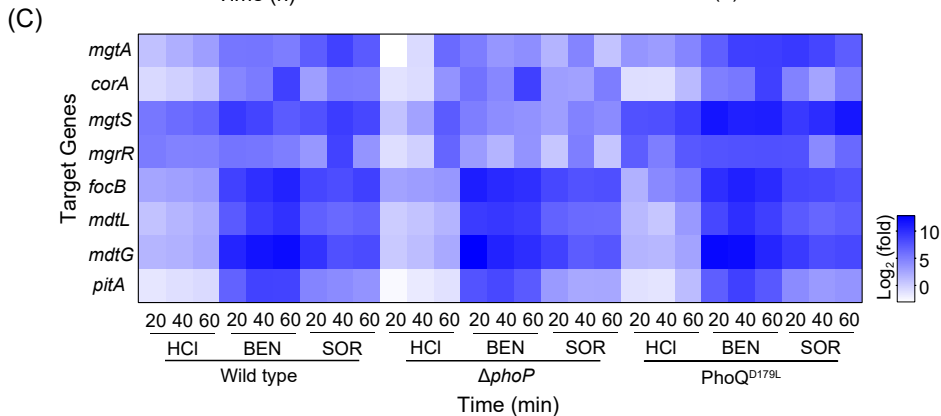

Supplement: Supplementary file 9 — Fig‐S9. [file MLF2-4-423-s002.pdf]

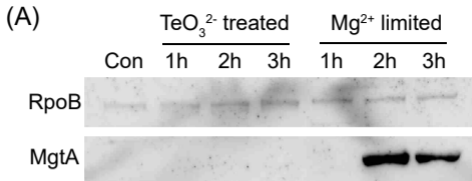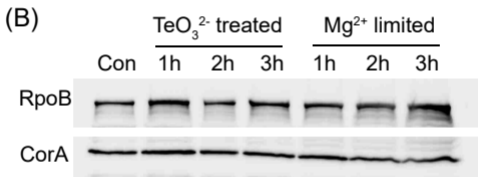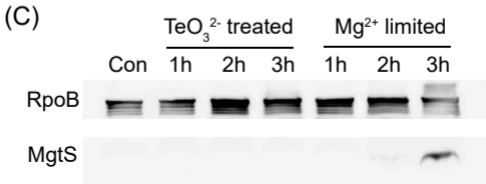

Supplement: Supplementary file 10 — Fig‐S10. [file MLF2-4-423-s003.pdf]

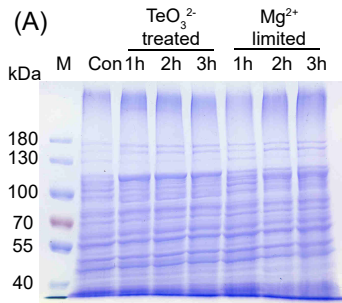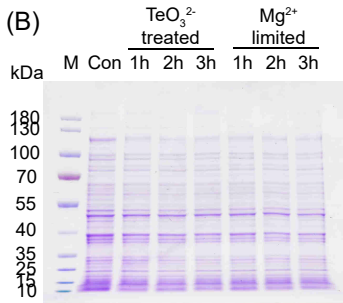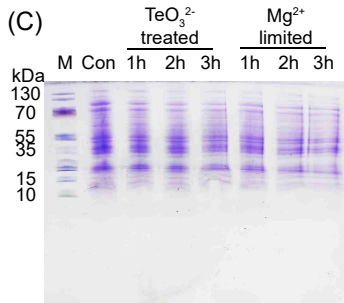

Supplement: Supplementary file 11 — Fig‐S11. [file MLF2-4-423-s013.pdf]

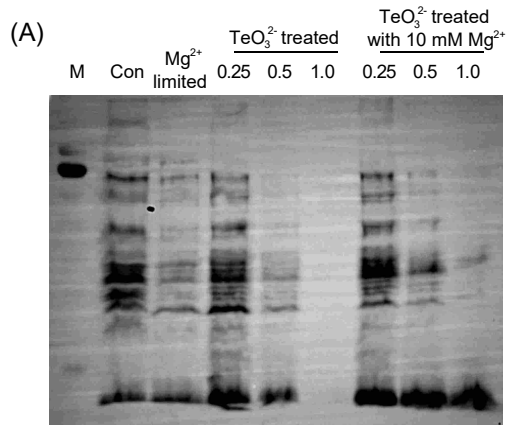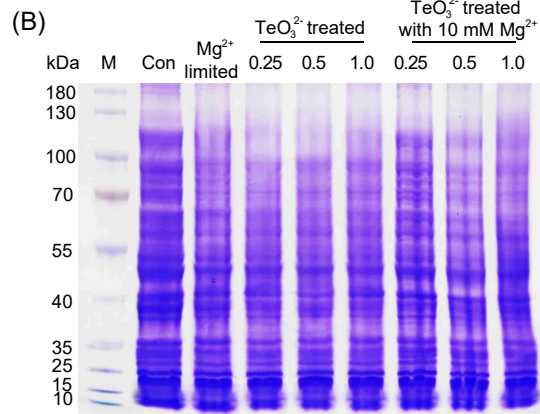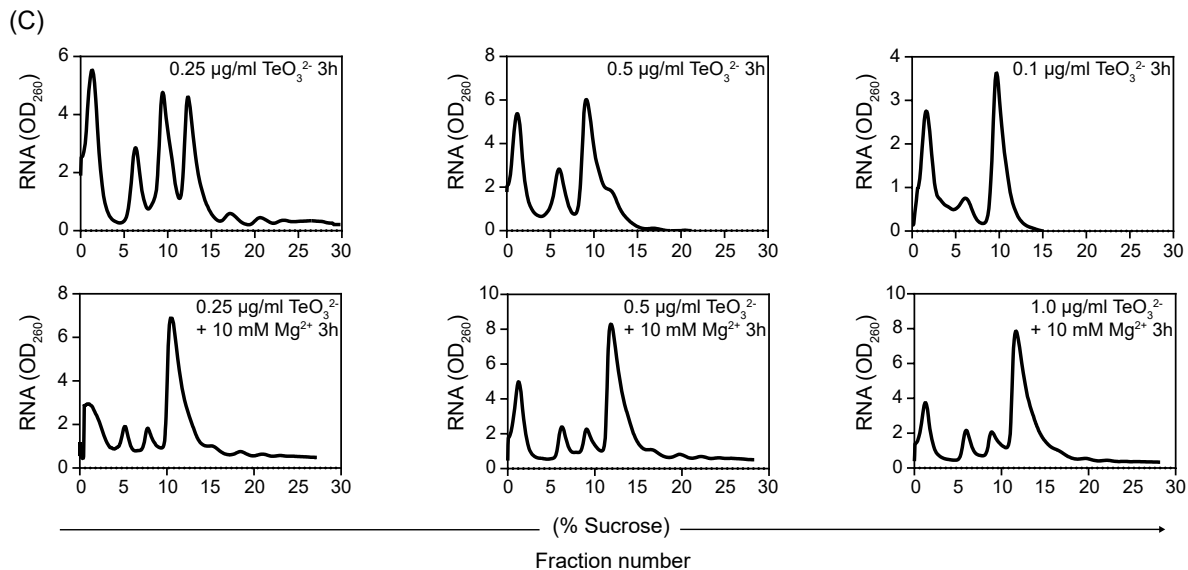

Supplement: Supplementary file 12 — Fig‐S12. [file MLF2-4-423-s005.pdf]

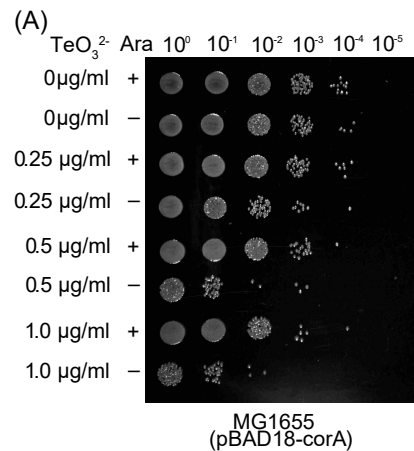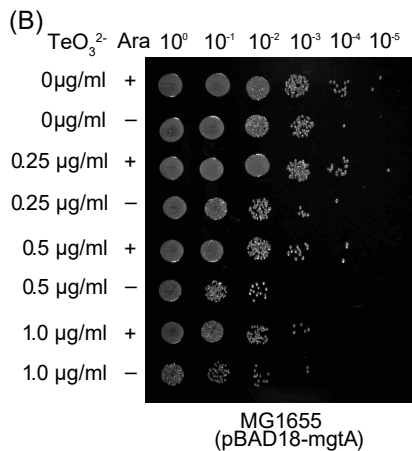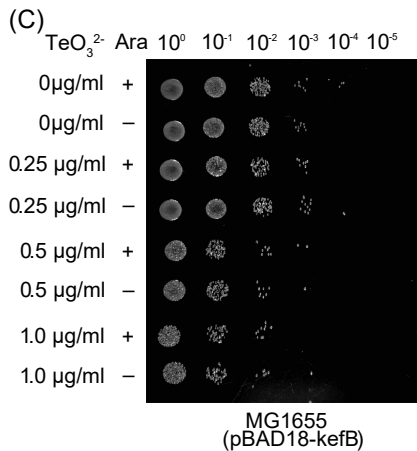

Supplement: Supplementary file 13 — Fig‐S13. [file MLF2-4-423-s015.pdf]

(A)

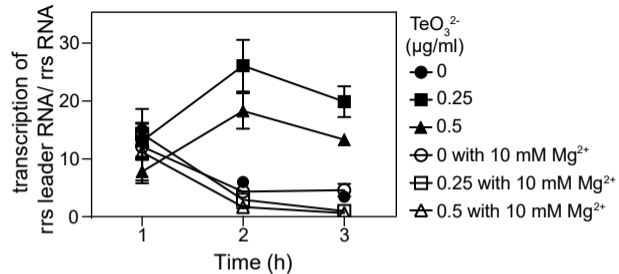

(B)

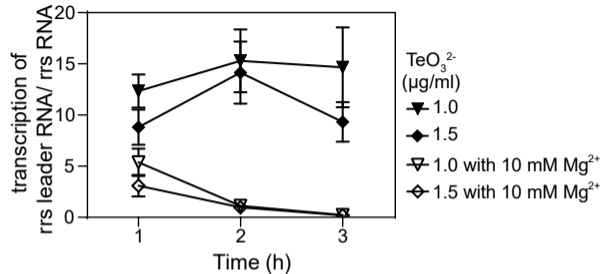

(C)

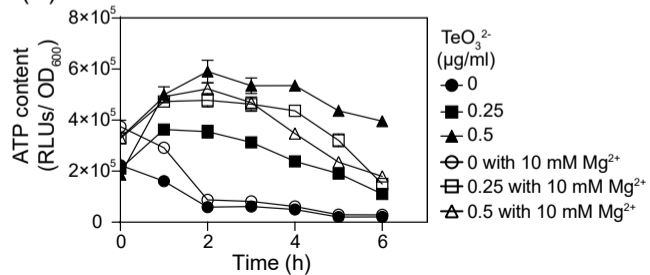

(D)

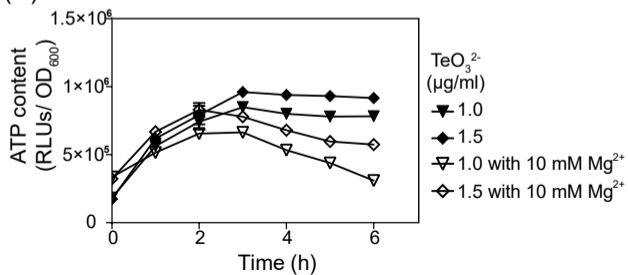

Supplement: Supplementary file 14 — Fig‐S14. [file MLF2-4-423-s021.pdf]

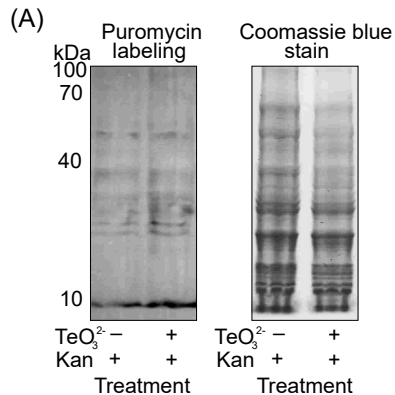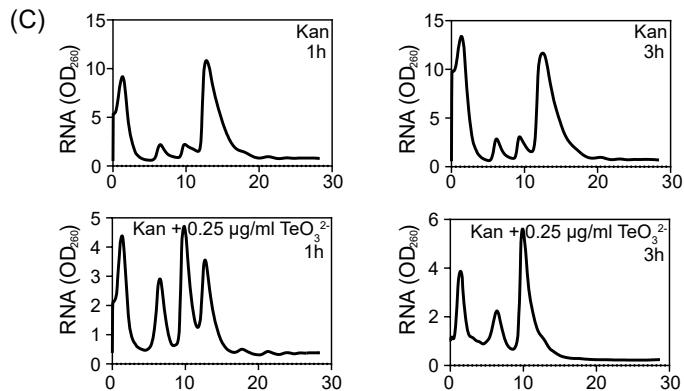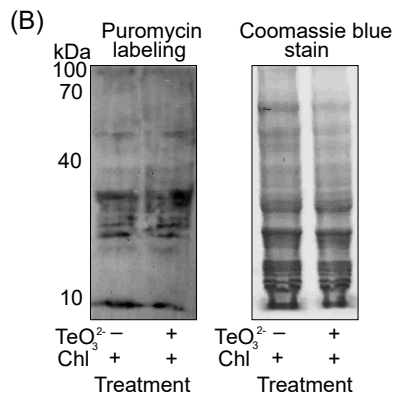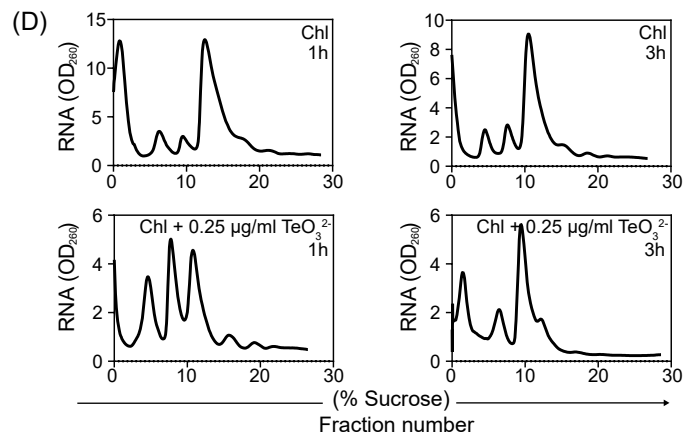

Supplement: Supplementary file 15 — Fig‐S15. [file MLF2-4-423-s019.pdf]

(A)

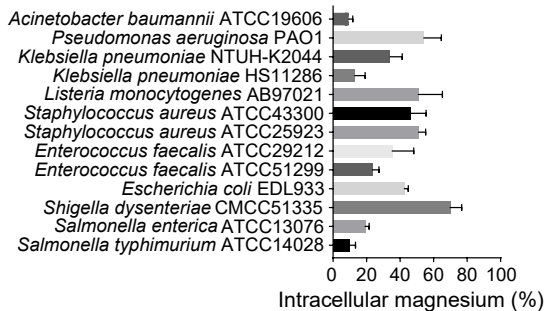

(B)

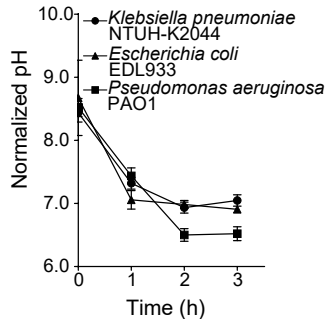

Supplement: Supplementary file 16 — Fig‐S16. [file MLF2-4-423-s001.pdf]

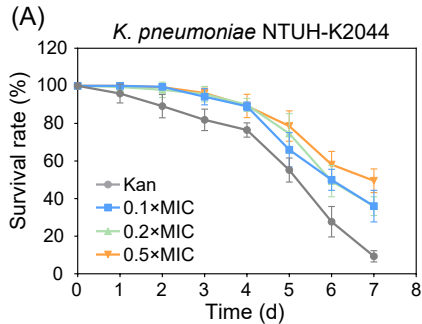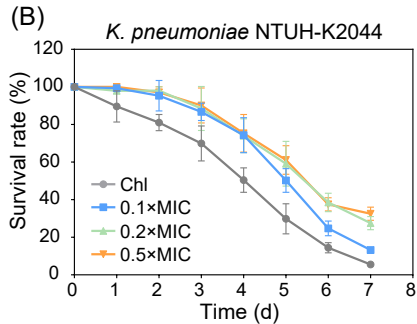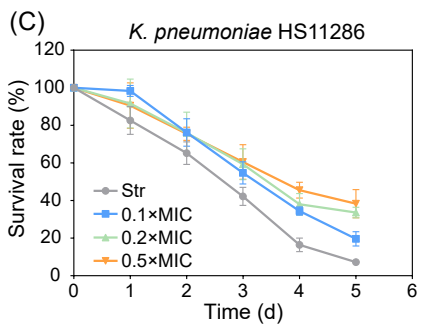

Supplement: Supplementary file 17 — Fig‐S17. [file MLF2-4-423-s018.pdf]

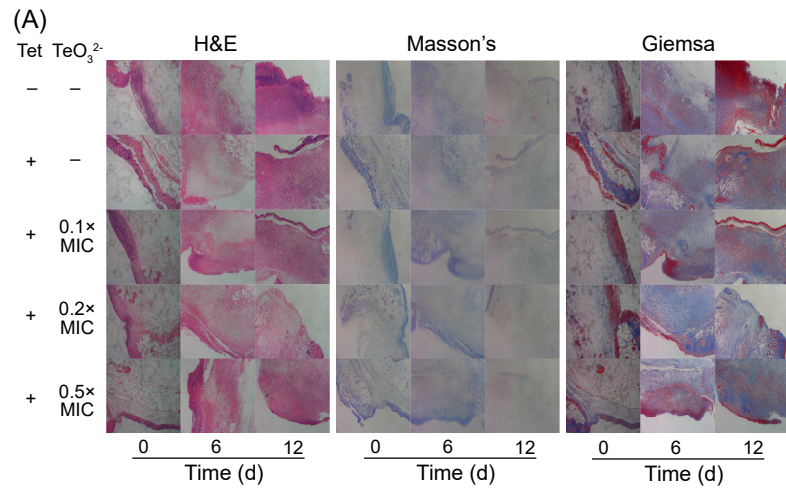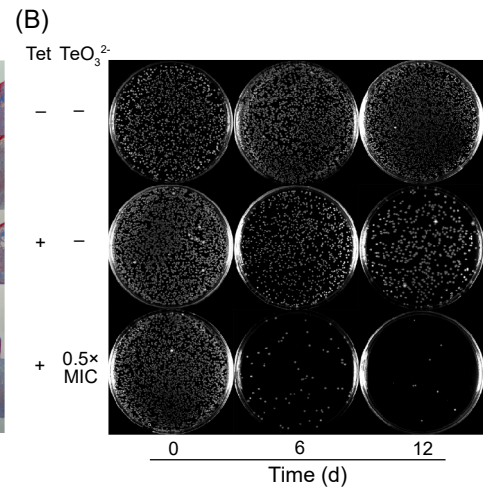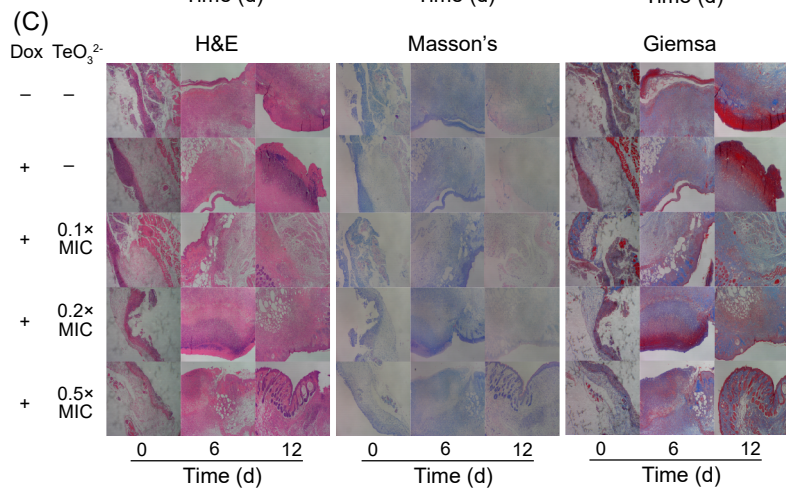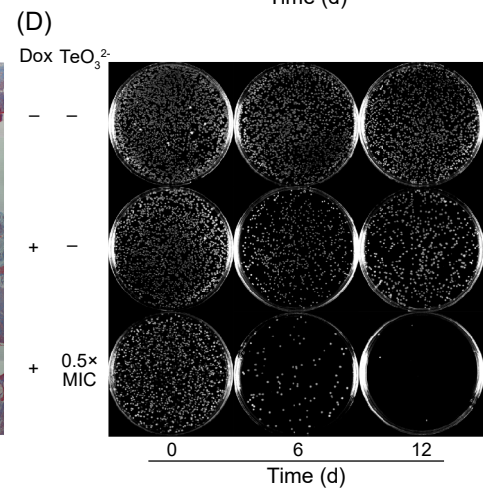

Supplement: Supplementary file 18 — Fig‐S18. [file MLF2-4-423-s007.pdf]

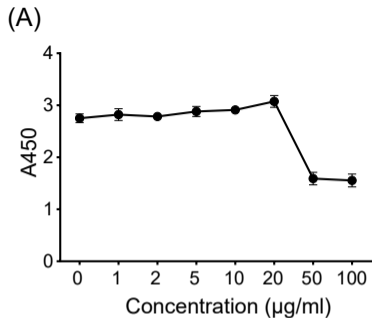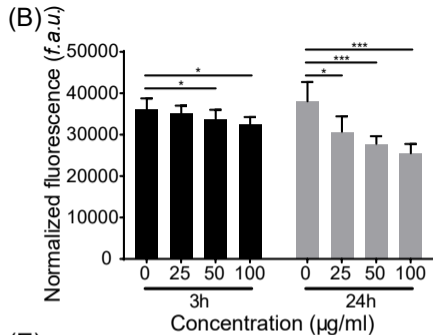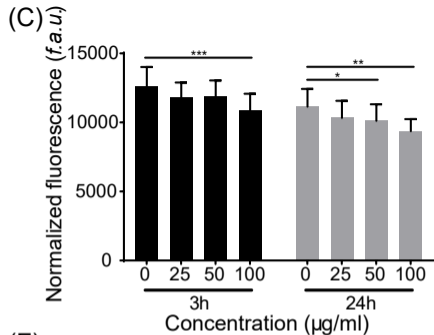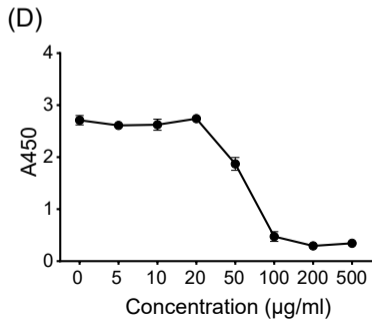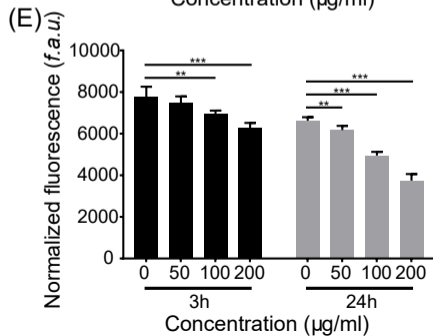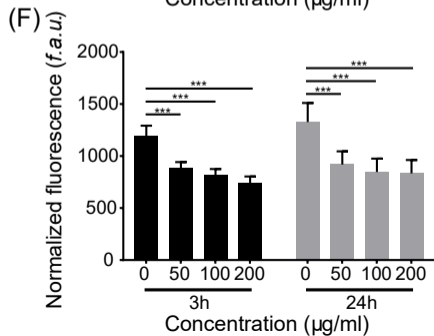

Supplement: Supplementary file 19 — Fig‐S19. [file MLF2-4-423-s012.pdf]

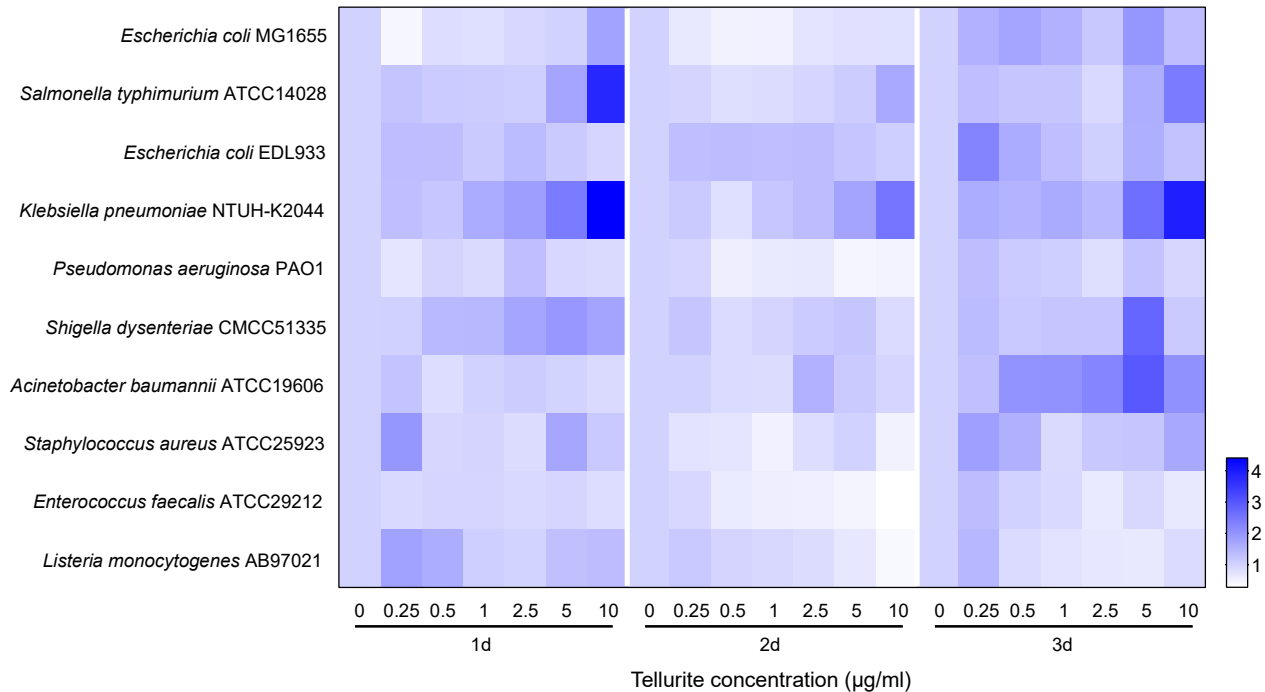

Supplement: Supplementary file 20 — Fig‐S20. [file MLF2-4-423-s016.pdf]
