# Supplementary material for: The antibacterial effect of tellurite is achieved through intracellular acidification and magnesium disruption
Source: mLife. 2025 Aug 24;4(4):423–36. doi: 10.1002/mlf2.70028 (PMC12395589; doi:10.1002/mlf2.70028)
Supplement: Supplementary file 21 — 20250318‐Supplementary material. [file MLF2-4-423-s020.docx]

**The antibacterial effect of tellurite is achieved through intracellular acidification and magnesium depletion**

Wanli Peng^1^, Yali Fu^1^, Yanqiu Wang^1^, Zixin Deng^1^, Daijie Chen^2^, Shuangjun Lin^1, 3^*****, Rubing Liang^1^*****

^1^ State Key Laboratory of Microbial Metabolism, Joint International Research Laboratory of Metabolic & Developmental Sciences, School of Life Sciences and Biotechnology, Shanghai Jiao Tong University, 800 Dongchuan Road, Shanghai 200240, China

^2^ State Key Laboratory of Microbial Metabolism, School of Pharmaceutical Sciences, Shanghai Jiao Tong University, 800 Dongchuan Road, Shanghai 200240, China

^3^ Frontiers Science Center for Transformative Molecules, Shanghai Jiao Tong University, 800 Dongchuan Road, Shanghai 200240, China

Corresponding author: Shuangjun Lin^1, 3^*****, Rubing Liang^1^*****

**Email:**

Shuangjun Lin: [linsj@sjtu.edu.cn,](mailto:linsj@sjtu.edu.cn,) and Rubing Liang: [icelike@sjtu.edu.cn](mailto:icelike@sjtu.edu.cn)

**Fig S1. Tellurite Treatment and Mg²⁺ Limitation Induce Distinct Transcriptional Responses in ROS and Ribosome-Associated Genes.** The transcriptional levels of genes associated with ROS reaction and ribosome assembly in *E. coli* MG1655 cultivated in M9 medium with tellurite (0.25 and 0.5 μg/ml) or Mg^2+^ limited medium were detected at 20‒60 min, and normalized by that of *gapA* gene (n=5; mean ± SD).

**Fig S2. Tellurite Treatment Disrupts Cellular pH Homeostasis and Inhibits Growth in *E. coli* MG1655.** (A) The pH variation in *E. coli* MG1655 cells under tellurite treatments. The normalized fluorescence intensity (pHluorin/mCherry) in *E. coli* MG1655 cells cultured in M9 medium (gray) or M9 medium supplied with tellurite (0.5 μg/ml, pH 7.0, red) was determined after 30 min and the results were plotted (n=5; mean ± SD). (B) The pH change in *E. coli* MG1655 cells treated by tellurite with the supplement of glutamate or arginine. The normalized fluorescence intensity (pHluorin/mCherry) was determined from 0 h to 12 h in *E. coli* MG1655 cells cultured in M9 medium (gray), M9 medium supplied with sodium glutamate (40 mM, pH 7.0; orange), or arginine (40 mM, pH 7.0; green) and treated with 0 μg/ml (circle), 0.25 μg/ml (square) or 0.5 μg/ml (rectangle) tellurite. The time course was quantified (n=3; mean ± SD).

**Fig S3. Selenite and Silver Ions Disrupt Cellular pH, Drain Magnesium, and Hamper Ribosome Assembly.** (A) The pH change in *E. coli* MG1655 cells under different treatments. The intracellular pH in *E. coli* MG1655 cells cultured in the medium supplied with tellurite (0.5 μg/ml), selenite (0.5 μg/ml) or AgNO_3_ (24 mM) were detected at 1-3 h and plotted (n=5; mean ± SD). (B) The change of total magnesium in *E. coli* MG1655 cells under AgNO_3_. The cellular contents of total magnesium in *E. coli* MG1655 cultured in the medium supplied with AgNO_3_ (24 mM) were quantified by ICP‒MS analysis at 1 h and 3 h. The metal contents were normalized by total protein amounts (n=5; mean ± SD; **p* < 0.05; ***p* < 0.01; *** *p* < 0.001). (C) The ribosome polysome analysis of *E. coli* MG1655 cells under selenite treatment. The polysome analyses were monitored in cells cultured in the medium with selenite (0.5 and 1.0 μg/ml) at 1 h and 3 h. The polysome profiles are the representative of four independent experiments. (D) The ribosome polysome analysis of *E. coli* MG1655 cells under AgNO_3_ treatment. The polysome analyses were monitored in cells cultured in the medium with AgNO_3_ (24 mM) with/without 10 mM Mg^2+^ at 1 h. The polysome profiles are the representative of four independent experiments.

**Fig S4. Tellurite Treatment Alters Metal Ion Homeostasis in *E. coli* MG1655 Cells.** The cellular contents of total manganese (A), cytoplasmic manganese (B), total calcium (C), total iron (D), total tellurium (E) and cytoplasmic tellurium (F) in *E. coli* MG1655 cells were determined. The cells treated with tellurite (0.25‒1.0 μg/ml) for 0‒3 h were quantified via ICP‒MS analysis and plotted after normalization to the total protein concentration (n=5; mean ± SD; **p* < 0.05; ***p* < 0.01; *** *p* < 0.001).

**Fig S5. Cell Morphology of *E. coli* MG1655 Remains Unchanged under Tellurite Treatment.** Photographs of the transmission electron micrographs (TEM, A) and the scanning electron micrographs (SEM, B) of *E. coli* MG1655 cells under different tellurite treatment (i to vii: 0 μg/ml, 0.25 μg/ml for 1 h, 0.25 μg/ml for 3 h, 0.5 μg/ml for 1 h, 0.5 μg/ml for 3 h, 1.0 μg/ml for 1 h, 1.0 μg/ml for 3 h) were shown. All the Photographs are representative of three independent experiments.

**Fig S6. Exogenous Mg²⁺ Mitigate Tellurite-Induced Growth Inhibition in *E. coli* MG1655.** The *E. coli* MG1655 cells were cultured in M9 media supplied with tellurite (0.5 μg/ml, 1.5 μg/ml) and different concentrations of Mg^2+^ (A-F, 0-100 mM) or Mn^2+^ (G-L, 0-500 μM). Cell growth (serial plating and OD_600_) was monitored and the results were plotted (n=5; mean ± SD).

**Fig S7. Tellurite Treatment Induces Differential Transcription of Transporter Genes in *E. coli* MG1655.** The transcription levels of different transporter genes in *E. coli* MG1655 cultured in medium supplied with tellurite (0.25, 0.5 μg/ml) or Mg^2+^ limited medium were detected at 20‒60 min, and normalized by that of *gapA* gene (n=4; mean ± SD).

**Fig S8. Tellurite Treatment Alters Cellular pH and Magnesium Contents in Different Strains.** (A) The pH change in different strains under tellurite treatments. The intracellular pH in wild-type and six mutants (∆*mdtL*, ∆*mdtG*, ∆*marB*, ∆*alx*, ∆*arsB*, ∆*rcnA*) cultured in medium supplied with 0.5 μg/ml tellurite were detected at 0‒3 h and plotted (n=5; mean ± SD). (B) The contents of total magnesium in four mutant strains under tellurite treatments. The contents of total magnesium in four mutant strains (∆*marB*, ∆*alx*, ∆*arsB*, ∆*rcnA*) treated with tellurite (0.5 μg/ml) for 0‒3 h were quantified by ICP-MS analysis and normalized by total protein amounts (n=5; mean ± SD; **p* < 0.05; ***p* < 0.01; *** *p* < 0.001).

**Fig S9. Various Acids affect Cellular pH, Magnesium Content, and the Transcription of Transporter in *E. coli* MG1655.** (A) The change of the total magnesium contents in *E. coli* MG1655 treated by tellurite and acids. The total magnesium content in *E. coli* MG1655 cells grown in M9 medium supplied with HCl (pH 3.0), sodium benzoate (pH 6.0) or potassium sorbate (pH 6.0) for 1 h or 3 h was quantified via ICP‒MS analysis and subsequently normalized to the total protein concentration (n=5; mean ± SD; **p* < 0.05; ***p* < 0.01; *** *p* < 0.001). (B) The pH variation in *E. coli* MG1655 treated by tellurite and acids. The intracellular pH of *E. coli* MG1655 cells cultured as described above was determined at 1‒3 h and plotted (n=5; mean ± SD). (C) The transcription levels of transporter genes in *E. coli* MG1655 and phoP/Q mutants treated with acids. The transcription levels of six transporter genes and two transport-associated genes in the wild-type, Δ*phoP* and PhoQ^D179L^ strains cultured in medium supplied with HCl (pH 3.0), sodium benzoate (pH 6.0) or potassium sorbate (pH 6.0) were determined at 20‒60 min and normalized to that of the *gapA* gene (n=5; mean ± SD; **p* < 0.05; ***p* < 0.01; *** *p* < 0.001).

**Fig S10. Western Blotting Reveals the Expression of Mg²⁺ Transport-Associated Proteins Varied under Stress Circumstances.** The *E. coli* MG1655 cells cultured in M9 medium supplied with tellurite (0.5 μg/ml) or Mg^2+^-limited medium were collected, and the expression of MgtA (A), CorA (B) and MgtS (C) was determined by Western blotting, with RpoB serving as an internal reference. All the gels are representative of four independent experiments.

**Fig S11. The SDS-PAGE Gel for Western Blotting Analysis Showed the Proteins Associated with Mg²⁺ Transportation.** The *E. coli* MG1655 cells cultured in medium with 0.5 μg/ml tellurite or Mg^2+^ limited medium were collected. The cells lyses were detected by SDS-PAGE and used for western blotting analysis proteins MgtA (A), CorA (B) and MgtS (C). The gels are representative of four independent experiments.

**Fig S12. Tellurite and Mg²⁺ Influence Protein Synthesis and Ribosome Assembly in *E. coli* MG1655.** The *E. coli* MG1655 cells for puromycin labeling analysis were cultured in Mg^2+^-limited medium, medium with tellurite (0.25-1.0 μg/ml) and medium with tellurite and 10 mM Mg^2+^ for 3 h. The Western blotting (A), SDS-PAGE (B) images and the polysome profiles (C) of the cultures were shown. The gels are representative of four independent experiments.

**Fig S13. Overexpression of Magnesium Transporters Promoptes Cell Survival of *E. coli* MG1655 Treated with Tellurite.** Three transporter genes (*mgtA*, *corA* and *kefB*) were cloned and inserted into pBAD18, which was subsequently induced with 10 mM L-arabinose for expression. *E. coli* MG1655 cells with or without MgtA (A), CorA (B) or KefB (C) overexpression were treated with tellurite (0-1.0 μg/ml) for 3 h. Cellular growth was monitored via serial plating at 10-fold dilutions (n=4; mean ± SD).

**Fig S14.Tellurite Enhances the Transcription of rRNA genes and Cellular ATP Contents in *E. coli* MG1655.** The transcription of rRNA genes in *E. coli* MG1655 treated with tellurite and/or Mg^2+^. (A, B) The transcription levels of *rrs* leader RNA to *rrs* RNA in *E. coli* MG1655 cells cultured in medium containing tellurite (0‒1.5 μg/ml) with/without 10 mM Mg^2+^ were detected. The transcription level of genes were normalized by *gapA* gene and the transcription ratio was calculated (n = 4; mean ± SD). The cellular ATP contents in *E. coli* MG1655 treated with tellurite and/or Mg^2+^. (C, D) The cellular ATP contents in these cells were also detected, and the RLUs were normalized by the OD_600_ value (n = 4; mean ± SD).

**Fig S15. Combination of Tellurite and Antibiotics Disrupts Ribosome Assembly and Inhibits Protein Synthesis in *E. coli* MG1655.** The protein expression of *E. coli* MG1655 in different treatments. Western blotting (left) and SDS‒PAGE (right) images of *E. coli* MG1655 cultured in media supplied with kanamycin (0.15 μg/ml, 0.5×MIC) (A) or chloramphenicol (0.6 μg/ml, 0.5×MIC) (B) and supplied with/without tellurite (0.25 μg/ml, 0.5×MIC) are shown. The gels are representative of five independent experiments. (C) The ribosome polysome analyses of *E. coli* MG1655 under different treatments. Polysome analyses were performed for cells cultured in M9 medium supplied with kanamycin (0.15 μg/ml, 0.5×MIC) or chloramphenicol (0.6 μg/ml, 0.5×MIC) and supplied with/without tellurite (0.25 μg/ml, 0.5×MIC) for 1 h and 3 h. The polysome profiles are representative of five independent experiments.

**Fig S16. Tellurite Alters Magnesium Content and Cellular pH in Various Pathogenic Strains.** (A) The contents of total magnesium in different pathogenic strains treated by tellurite. The total magnesium content in different pathogenic strains grown in M9 medium supplemented with tellurite (0.5 × MIC) for 3 h was quantified via ICP‒MS analysis and compared with that in untreated cells to calculate the relative fold changes. The metal concentrations were normalized to the total protein concentration (n=4; mean ± SD). (B) The cellular pH in different pathogenic strains treated by tellurite. The intracellular pH of the three pathogenic bacterial strains grown in M9 medium supplemented with tellurite at the MIC was determined at 0-3 h and plotted (n=5; mean ± SD).

**Fig S17. The Tellurite-Antibiotic Combination Therapy Effectively Treats Pathogen-Infected *C. elegans.*** The *C. elegans* infected by strain *K. pneumoniae* NTUH-K2044 or HS11286 treated by different combination of tellurite and anytibiotics. *C. elegans* infected with *K. pneumoniae* NTUH-K2044 were treated with kanamycin (5 μg/ml, 2 × MIC) (A) or chloramphenicol (250 μg/ml, 2 × MIC) (B) with or without tellurite (0.1‒0.5 × MIC). The survival rates were monitored weekly and are plotted (n=4; mean ± SD). (C) *C. elegans* infected with *K. pneumoniae* HS11286 were treated with streptomycin (15 μg/ml, 2 × MIC) with or without tellurite (0.1‒0.5 × MIC), after which the survival rates were monitored and the results were plotted (n=4; mean ± SD).

**Fig S18. The Combination Therapy Reduces Bacterial Load and Improves Tissue Integrity in Mice.** (A) The histological analysis of skin wounds in mice infected with *K. pneumoniae* NTUH-K2044. Histological analysis of skin wounds in mice infected with *K. pneumoniae* NTUH-K2044 and treated with tetracycline (5 μg/ml, 2×MIC) alone or in combination with tellurite (0.1‒0.5 × MIC) for six or twelve days was performed. The photographs are representative of four independent experiments. (B) The plate assay of *K. pneumoniae* NTUH-K2044 in the skin wounds in mice in different treatments. Plating assays were performed using cells from the skin wounds of mice infected with *K. pneumoniae* NTUH-K2044 and treated with tetracycline alone (5 μg/ml, 2 × MIC) or in combination with tellurite (0.5 × MIC) for six or twelve days. The photographs are representative of four independent experiments. (C) The histological analysis of skin wounds in mice infected with *K. pneumoniae* HS11286. The histological analysis shows the skin wounds in mice infected with *K. pneumoniae* HS11286 treated with doxycycline (100 μg/ml, 2 × MIC) alone or in combination with tellurite (0.1-0.5 × MIC) for six or twelve days. The photographs are representative of three independent experiments. (D) The plate assay of *K. pneumoniae* HS11286 in the skin wounds in mice under different treatments. The plating assays were performed using cells from the skin wounds of mice infected with *K. pneumoniae* HS11286 treated with doxycycline (100 μg/ml, 2 × MIC) alone or in combination with tellurite (0.5 × MIC) for six or twelve days. The photographs are representative of four independent experiments.

**Fig S19. Tellurite Disrupts the Homeostasis of Intracellular pH and Magnesium in Eukaryotic Cells.** The MIC of tellurite, cellular pH and intracellular magnesium in Hela CELLS under tellurite treatment were determined. The MIC of tellurite to Hela cells (A) and MCF7 cells (D) were determined with CCK-8 method using tellurite of 1‒200 μg/ml. The fluorescence intensity (BCECF, AM) was determined to analyze cellular pH in Hela cells (B) and MCF7 cells (E) when treated with tellurite. The fluorescence intensity (Mag-Fluo-4, AM) was determined to analyze intracellular magnesium in HeLa cells (C) and MCF7 cells (F) when treated with tellurite. (n = 6; mean ± SD).

**Fig. S20. Tellurite Affected Biofilm Formation in Diverse Bacterial Species.** Biofilm formation by different bacterial cells under tellurite treatment was assessed using crystal violet staining. The contents of biofilm were quantified as the ratio of OD_595_ to OD_600_ (biofilm content to bacterial density). The results were normalized to the control group without tellurite treatment. Measurements were conducted at 1, 2, and 3 days (n = 6; mean ± SD).

**Table S1. Proteomics data of some stress-related proteins**

| **Proteins** | **Control** | **TeO_3_^2-^ treated 1h** | | **TeO_3_^2-^ treated 2h** | **TeO_3_^2-^ treated 3h** | **Mg^2+^ limited 1h** | **Mg^2+^ limited 2h** | **Mg^2+^ limited 3h** |
| --- | --- | --- | --- | --- | --- | --- | --- | --- |
| Hcr | 1 | | 1.08 | 0.54 | 0.67 | 0.90 | 0.94 | 0.77 |
| SodC | 1 | | 0.89 | 0.79 | 0.98 | 1.09 | 3.64 | 4.42 |
| SodB | 1 | | 0.77 | 0.73 | 0.64 | 1.36 | 0.98 | 0.99 |
| SodA | 1 | | 0.84 | 0.66 | 0.67 | 0.87 | 0.58 | 0.53 |
| XdhD | ND | | ND | ND | ND | ND | ND | ND |
| KatE | 1 | | 1.37 | 1.09 | 0.92 | 2.22 | 3.84 | 3.41 |
| TrxB | 1 | | 0.59 | 0.60 | 0.62 | 0.73 | 0.60 | 0.60 |
| TrxC | 1 | | 0.91 | 0.90 | 0.90 | 1.07 | 0.86 | 0.81 |
| TrxA | 1 | | 0.96 | 0.95 | 0.96 | 1.00 | 1.06 | 1.00 |
| BtuE | 1 | | 1.02 | 0.83 | 0.89 | 0.90 | 0.77 | 0.73 |
| OsmC | 1 | | 1.03 | 1.20 | 1.34 | 0.82 | 2.41 | 2.94 |
| RpsT | ND | | ND | ND | ND | ND | ND | ND |
| RpsO | 1 | | 1.01 | 1.05 | 0.94 | 1.18 | 0.86 | 0.80 |
| PrlI | ND | | ND | ND | ND | ND | ND | ND |
| RpmB | 1 | | 0.83 | 0.88 | 0.83 | 0.77 | 0.73 | 0.76 |
| RplU | ND | | ND | ND | ND | ND | ND | ND |
| RpmL | ND | | ND | ND | ND | ND | ND | ND |
| MgtS | ND | | ND | ND | ND | ND | ND | ND |
| PhoP | 1 | | 1.21 | 1.11 | 1.03 | 1.51 | 2.74 | 3.18 |
| PhoQ | 1 | | 2.10 | 2.41 | 2.36 | 2.16 | 4.95 | 6.77 |
| RcnA | ND | | ND | ND | ND | ND | ND | ND |
| MarB | ND | | ND | ND | ND | ND | ND | ND |
| Alx | ND | | ND | ND | ND | ND | ND | ND |
| MarA | ND | | ND | ND | ND | ND | ND | ND |
| ArsB | ND | | ND | ND | ND | ND | ND | ND |
| EntS | 1 | | 0.71 | 0.51 | 0.44 | 0.83 | 1.13 | 0.95 |
| AcrB | 1 | | 1.00 | 0.93 | 0.87 | 0.83 | 0.75 | 0.77 |
| MntP | ND | | ND | ND | ND | ND | ND | ND |
| MdtL | ND | | ND | ND | ND | ND | ND | ND |
| EmrE | ND | | ND | ND | ND | ND | ND | ND |
| YbhF | 1 | | 1.47 | 0.97 | 1.23 | 0.65 | 1.45 | 1.15 |
| Bcr | 1 | | 1.12 | 1.31 | 1.79 | 0.86 | 1.23 | 1.02 |
| MdtG | ND | | ND | ND | ND | ND | ND | ND |
| FocB | ND | | ND | ND | ND | ND | ND | ND |
| EmrB | ND | | ND | ND | ND | ND | ND | ND |
| EmrY | ND | | ND | ND | ND | ND | ND | ND |
| MdtJ | ND | | ND | ND | ND | ND | ND | ND |
| FocA | ND | | ND | ND | ND | ND | ND | ND |

ND: not detected

**Table S2. Transporter and regulator genes detected in this work**

| **Genes** | **Description** | **Location** |
| --- | --- | --- |
| *mgtA* | Mg(2(+)) importing P-type ATPase | NC_000913.3 (4467625..4470321) |
| *corA* | Ni(2(+))/Co(2(+))/Mg(2(+)) transporter | NC_000913.3 (4001426..4002376) |
| *kefB* | K(+) : H(+) antiporter KefB | NC_000913.3 (3478802..3480607) |
| *phoP* | DNA-binding transcriptional dual regulator PhoP | NC_000913.3 (1189776..1190447) |
| *phoQ* | sensor histidine kinase PhoQ | NC_000913.3 (1188316..1189776) |
| *pitA* | metal phosphate:H(+) symporter PitA | NC_000913.3 (3637642..3639141) |
| *focB* | formate channel FocB | NC_000913.3 (2613934..2614782) |
| *mdtL* | efflux pump MdtL | NC_000913.3 (3891615..3892790) |
| *mdtG* | efflux pump MdtG | NC_000913.3 (1114264..1115490) |
| *alx* | putative membrane-bound redox modulator Alx | NC_000913.3 (3238580..3239545) |
| *marB* | multiple antibiotic resistance protein MarB | NC_000913.3 (1619989..1620207) |
| *arsB* | arsenite/antimonite:H(+) antiporter | NC_000913.3 (3648935..3650224) |
| *rcnA* | Ni(2(+))/Co(2(+)) exporter | NC_000913.3 (2185917..2186741) |
| *marA* | NA-binding transcriptional dual regulator MarA | NC_000913.3 (1619574..1619957) |
| *entS* | enterobactin exporter EntS | NC_000913.3 (622300..623550) |
| *acrB* | multidrug efflux pump RND permease AcrB | NC_000913.3 (481254..484403) |
| *mntP* | Mn(2(+)) exporter | NC_000913.3 (1905688..1906254) |
| *emrE* | multidrug/betaine/choline efflux transporter EmrE | NC_000913.3 (568315..568647) |
| *ybhF* | ABC exporter ATP binding subunit YbhF | NC_000913.3 (827245..828981) |
| *bcr* | multidrug efflux pump Bcr | NC_000913.3 (2278570..2279760) |
| *emrB* | multidrug efflux pump membrane subunit EmrB | NC_000913.3 (2812616..2814154) |
| *emrY* | tripartite efflux pump membrane subunit EmrY | NC_000913.3 (2480638..2482176) |
| *mdtJ* | multidrug/spermidine efflux pump membrane subunit MdtJ | NC_000913.3 (1673136..1673501) |
| *focA* | formate channel FocA | NC_000913.3 (953609..954466) |

**Table S3. Fractional inhibitory concentration (FIC) index of tellurite and traditional antibiotics.**

| **Strain** | **Kanamycin** | **Nalidixic acid** | **Polymyxin B** | **Erythromycin** | **Ampicillin** | **Vancomycin** | **Tetracycline** | **Rifampicin** | **Chloramphenicol** | **Sulfanilamide** |
| --- | --- | --- | --- | --- | --- | --- | --- | --- | --- | --- |
| *E.coli* MG1655 | 0.63 | 0.67 | 0.87 | 1.51 | 1.0 | 1.33 | 0.36 | 0.92 | 0.41 | 0.38 |
| *S. aureus* ATCC25923 | 0.33 | 0.5 | 1.67 | 1.0 | 1.09 | 1.67 | 0.94 | 0.32 | 0.59 | 1.68 |

**Table S4. MIC of tellurite and antibiotics to different bacterial strains**

| **Strain** | **Tellurite**  **(μg/ml)** | **Tetracycline**  **(μg/ml)** | **Kanamycin**  **(μg/ml)** | **Chloramphenicol**  **(μg/ml)** |
| --- | --- | --- | --- | --- |
| *Escherichia coli* MG1655 | 0.5 | 0.625 | 0.31 | 1.25 |
| *Staphylococcus aureus* ATCC25923 | 62.5 | 0.25 | 0.125 | 2.5 |
| *Salmonella typhimurium* ATCC14028 | 0.2 | 0.625 | 2.5 | 2.5 |
| *Escherichia coli* O157:H7 EDL933 | 0.8 | 0.625 | 10 | 2.5 |
| *Klebsiella pneumoniae* NTUH-K2044 | 0.2 | 2.5 | 10 | 5 |
| *Pseudomonas aeruginosa* PAO1 | 62.5 | 2.5 | 1.25 | 1.25 |
| *Shigella dysenteriae* CMCC51335 | 25 | 0.08 | 0.08 | 0.08 |
| *Acinetobacter baumannii* ATCC19606 | 1.6 | 1.25 | 0.625 | 50 |
| *Enterococcus faecalis* ATCC29212 | 500 | 12.5 | 3.125 | 6.25 |
| *Listeria monocytogenes* AB97021 | 250 | 0.156 | 0.05 | 3.125 |
| *Salmonella typhimurium* ATCC13076 | 0.1 | - | - | - |
| *Enterococcus faecalis* ATCC51299 | 500 | - | - | - |
| *Staphylococcus aureus* ATCC43300 | 250 | - | - | - |
| *Klebsiella pneumoniae* HS11286 | 0.4 | - | - | - |

**Table S5. Applications of various antibacterial agents against pathogens**

| **Antibacterial agents** | **Concentration** | **Applications** | **Reference** |
| --- | --- | --- | --- |
| Tellurite | 0.1 μg/ml *in vivo*, 0.2 ng/ml *in vitro* | Combination with traditional antibiotics against *K. pneumoniae* | This work |
| Silver Nanoparticles | 2×10^11^ particles/ml, ~0.85 μg/ml *in vitro* | Combination with Imipenem against *K. pneumoniae* | ^1^ |
| Silver Nanoparticles | 50 μg/ml *in vitro* | Antibacterial activity to *K. pneumoniae* | ^2^ |
| Zinc ions | 31.25 μg/ml *in vitro* | Antibacterial activity to *K. pneumoniae* | ^3^ |
| Silver ions | 10^-7^ M *in vitro* | Antibacterial activity to *E. coli* and *S. aureus* | ^4^ |
| Copper ions | 10^-5^ M *in vitro* | Antibacterial activity to *E. coli* and *S. aureus* | ^4^ |
| Zinc ions | 10^-5^ M *in vitro* | Antibacterial activity to *E. coli* and *S. aureus* | ^4^ |
| Quinones | 200-500 μg/ml *in vitro* | Antibacterial activity to *B.subtilis* *Proteus vulgaris* and *S. aureus* | ^5^ |
| Flavones | 7.81-15.62 µg/mL *in vitro* | Antibacterial activity to *P. aeruginosa* and *E. coli* | ^6^ |

**Table S6. Strains used in this study**

| **Strains** | **Description** | **Reference/Source** |
| --- | --- | --- |
| *Escherichia coli* MG1655 | Coli Genetic Stock Center strain (CGSC) No. 6300 | CGSC |
| *Escherichia coli* MG1655 Δ*pitA* | The *pitA*-deleted mutant of *E. coli* MG1655 | This study |
| *Escherichia coli* MG1655 Δ*focB* | The *focB*-deleted mutant of *E. coli* MG1655 | This study |
| *Escherichia coli* MG1655 Δ*mdtL* | The *mdtL*-deleted mutant of *E. coli* MG1655 | This study |
| *Escherichia coli* MG1655 Δ*mdtG* | The *mdtG*-deleted mutant of *E. coli* MG1655 | This study |
| *Escherichia coli* MG1655 Δ*rcnA* | The *rcnA*-deleted mutant of *E. coli* MG1655 | This study |
| *Escherichia coli* MG1655 Δ*alx* | The *alx*-deleted mutant of *E. coli* MG1655 | This study |
| *Escherichia coli* MG1655 Δ*marB* | The *marB*-deleted mutant of *E. coli* MG1655 | This study |
| *Escherichia coli* MG1655 Δ*arsB* | The *arsB*-deleted mutant of *E. coli* MG1655 | This study |
| *Escherichia coli* MG1655 Δ*phoP* | The *phoP*-deleted mutant of *E. coli* MG1655 | This study |
| *Escherichia coli* MG1655 *phoQ* ^D179L^ | Chromosomal mutation of the Leu residue D179 | Tao Dong Lab |
| *Klebsiella pneumoniae* NTUH-K2044 | Strain used for antibacterial experiment | Lab preserved |
| *Klebsiella pneumoniae* HS11286 | Strain used for antibacterial experiment | Hongyu Ou Lab |
| *Salmonella typhimurium* ATCC14028 | Strain used for antibacterial experiment | Lab preserved |
| *Salmonella enterica* ATCC13076 | Strain used for antibacterial experiment | Xianming Shi Lab |
| *Shigella dysenteriae* CMCC51335 | Strain used for antibacterial experiment | Xianming Shi Lab |
| *Escherichia coli* EDL933 | Strain used for antibacterial experiment | Qing Liu Lab, University of Shanghai for Science and Technology |
| *Acinetobacter baumannii* ATCC19606 | Strain used for antibacterial experiment | Daijie Chen Lab |
| *Enterococcus faecalis* ATCC51299 | Strain used for antibacterial experiment | Daijie Chen Lab |
| *Enterococcus faecalis* ATCC29212 | Strain used for antibacterial experiment | Xianming Shi Lab |
| *Staphylococcus aureus* ATCC25923 | Strain used for antibacterial experiment | Chunlei Shi Lab |
| *Staphylococcus aureus* ATCC43300 | Strain used for antibacterial experiment | Chunlei Shi Lab |
| *Listeria monocytogenes* AB97021 | Strain used for antibacterial experiment | Xianming Shi Lab |
| *Pseudomonas aeruginosa* PAO1 | Strain used for antibacterial experiment | Lab preserved |

**Table S7. Plasmids used in this study**

| **Plasmids** | **Description** | **Source/Reference** |
| --- | --- | --- |
| pACYC-Duet-1 | Vector with a p15A origin, Cm^R^ | Novagen |
| pA-P*_rrnB_*-*eGFP* | eGFP under 5’-UTR and promoter of *E. coli* MG1655 *rrnB*, rep_p15A_, Cm^R^ | This study |
| pBAD18 | Tightly controlled expression vectors regulated by the arabinose operon | ^7^ |
| pB-corA | The plasmid pBAD18 inserted with *corA* gene at the EcoR I/Hind III sites, Amp^R^ | This study |
| pB-mgtA | The plasmid pBAD18 inserted with *mgtA* gene at the EcoR I/Hind III sites, Amp^R^ | This study |
| pB-kefB | The plasmid pBAD18 inserted with *kefB* gene at the EcoR I/Hind III sites, Amp^R^ | This study |
| pB-marB | The plasmid pBAD18 inserted with *marB* gene at the EcoR I/Hind III sites, Amp^R^ | This study |
| pB-alx | The plasmid pBAD18 inserted with *alx* gene at the EcoR I/Hind III sites, Amp^R^ | This study |
| pB-arsB | The plasmid pBAD18 inserted with *arsB* gene at the EcoR I/Hind III sites, Amp^R^ | This study |
| pB-rcnA | The plasmid pBAD18 inserted with *rcnA* gene at the EcoR I/Hind III sites, Amp^R^ | This study |
| pB-mdtL | The plasmid pBAD18 inserted with *mdtL* gene at the EcoR I/Hind III sites, Amp^R^ | This study |
| pB-mdtG | The plasmid pBAD18 inserted with *mdtG* gene at the EcoR I/Hind III sites, Amp^R^ | This study |
| pB-focB | The plasmid pBAD18 inserted with *focB* gene at the EcoR I/Hind III sites, Amp^R^ | This study |
| pCas | Vector for genome editing, pSC101 origin, Km^R^ | ^8^ |
| pTargetF | Vector for genome editing, pMB1 origin, Sm^R^ | ^8^ |
| pT-marB | pTargetF plasmid harboring correspond sgRNA, with donor DNA fragment at the Hind III/Xho I sites, Sm^R^ | This study |
| pT-alx | pTargetF plasmid harboring correspond sgRNA, with donor DNA fragment at the Hind III/Xho I sites, Sm^R^ | This study |
| pT-arsB | pTargetF plasmid harboring correspond sgRNA, with donor DNA fragment at the Hind III/Xho I sites, Sm^R^ | This study |
| pT-rcnA | pTargetF plasmid harboring correspond sgRNA, with donor DNA fragment at the Hind III/Xho I sites, Sm^R^ | This study |
| pT-mdtL | pTargetF plasmid harboring correspond sgRNA, with donor DNA fragment at the Hind III/Xho I sites, Sm^R^ | This study |
| pT-mdtG | pTargetF plasmid harboring correspond sgRNA, with donor DNA fragment at the Hind III/Xho I sites, Sm^R^ | This study |
| pT-focB | pTargetF plasmid harboring correspond sgRNA, with donor DNA fragment at the Hind III/Xho I sites, Sm^R^ | This study |
| pT-mgtA-FLAG | pTargetF plasmid harboring correspond sgRNA, with donor DNA fragment at the Hind III/Xho I sites, Sm^R^ | This study |
| pT-corA-FLAG | pTargetF plasmid harboring correspond sgRNA, with donor DNA fragment at the Hind III/Xho I sites, Sm^R^ | This study |
| pT-mgtS-FLAG | pTargetF plasmid harboring correspond sgRNA, with donor DNA fragment at the Hind III/Xho I sites, Sm^R^ | This study |

**Table S8. Oligonucleotides used in this study**

| **Name** | **Sequences (5’-3’)** | **Description** |
| --- | --- | --- |
| pTarget-VF | GCCTTTTTACGGTTCCTGGC | Used for verification and sequence of pTargetF series plasmids |
| pTarget-VR | TCATAGCACGATCAACGGCA |  |
| marB-UF | agagtcgacctgcagaagcttTGCTTAAGAAAGTCCTGCCGT | Used for the construction of plasmid pT-marB |
| marB-UR | gccagaacaaaGATTGCCTCAGTGACGTTGTCA |  |
| marB-DF | gaggcaatcTTTGTTCTGGCCCCGACATC | Used for the construction of plasmid pT-marB |
| marB-DR | ggagctgcacatgaactcgagGATCATGTCGCACTCAACGC |  |
| marB-SPF | agtgataagtcggatgcgctgttttagagctagaaatagcaag | Used for the construction of plasmid pT-marB |
| marB-SPR | agcgcatccgacttatcacttagtattatacctaggactgagc |  |
| marB-VF | GTCGCATTGGTGTGGCTTTT | Used for the verification and sequence of MGΔmarB strain |
| marB-VR | ATGGGATCTGGGGGACGTTA |  |
| alx-UF | agagtcgacctgcagaagcttCAGAAAGTGTGCTTCGTGCC | Used for the construction of plasmid pT-alx |
| alx-UR | cggcttaggcagattaaaaaAGAAGTTCCTTACACATAAAAAAACGGC |  |
| alx-DF | TTTTTAATCTGCCTAAGCCGTGTACC | Used for the construction of plasmid pT-alx |
| alx-DR | ggagctgcacatgaactcgagGCGGGTTTTGAGATCCATGC |  |
| alx-SPF | tgtttaacgccgctttctgggttttagagctagaaatagcaag | Used for the construction of plasmid pT-alx |
| alx-SPR | ccagaaagcggcgttaaacatagtattatacctaggactgagc |  |
| alx-VF | CAGAAAGTGTGCTTCGTGCC | Used for the verification and sequence of MGΔalx strain |
| alx-VR | AATATCACCCGCGCTACTGG |  |
| arsB-UF | agagtcgacctgcagaagcttGCAACAATCAGGAGCGCAAT | Used for the construction of plasmid pT-arsB |
| arsB-UR | cagtatctcaAATGCCTCCCGGATAAAACACATC |  |
| arsB-DF | gggaggcattTGAGATACTGATATGAGCAACATTACCATT | Used for the construction of plasmid pT-arsB |
| arsB-DR | ggagctgcacatgaactcgagAGCGTCGCATCCGACATAAA |  |
| arsB-SPF | ggtccatccgggtgatattcgttttagagctagaaatagcaag | Used for the construction of plasmid pT-arsB |
| arsB-SPR | gaatatcacccggatggacctagtattatacctaggactgagc |  |
| arsB-VF | ACCAGCACGTCCTTGCAATA | Used for the verification and sequence of MGΔarsB strain |
| arsB-VR | CTACTGCGCTGTATCGCTGA |  |
| rcnA-UF | agagtcgacctgcagaagcttGACTTCCCTACGCTGGCATT | Used for the construction of plasmid pT-rcnA |
| rcnA-UR | gcaagcctaaaggattgagGATAATAATTCTTAGTATTAATTCGGCAATCTGAT |  |
| rcnA-DF | cCTCAATCCTTTAGGCTTGCATGTTAT | Used for the construction of plasmid pT-rcnA |
| rcnA-DR | ggagctgcacatgaactcgagATTTTACCGTCGGTGTCGCT |  |
| rcnA-SPF | ctgctcaatcagcagaaccggttttagagctagaaatagcaag | Used for the construction of plasmid pT-rcnA |
| rcnA-SPR | cggttctgctgattgagcagtagtattatacctaggactgagc |  |
| rcnA-VF | TCCAGCACCTTCAGAACGAC | Used for the verification and sequence of MGΔrcnA strain |
| rcnA-VR | GTTGCGGCGAAGTCAAAACT |  |
| mdtL-UF | agagtcgacctgcagaagcttACAGCACGGCATTATCGAGT | Used for the construction of plasmid pT-mdtL |
| mdtL-UR | cagattgagaGGGCTAAAGCGTCCTGATAGTAAG |  |
| mdtL-DF | gctttagcccTCTCAATCTGCTGCTTTGTCTGC | Used for the construction of plasmid pT-mdtL |
| mdtL-DR | ggagctgcacatgaactcgagACGCAGGGCACATCACTAAA |  |
| mdtL-SPF | cgcaggctgttgttacgtaggttttagagctagaaatagcaag | Used for the construction of plasmid pT-mdtL |
| mdtL-SPR | ctacgtaacaacagcctgcgtagtattatacctaggactgagc |  |
| mdtL-VF | CACAGCCATTTTCGTTGCCA | Used for the verification and sequence of MGΔmdtL strain |
| mdtL-VR | GTCACGTTTATCCGCTTCGC |  |
| mdtG-UF | agagtcgacctgcagaagcttTGCTGCCGATCGACATTCAT | Used for the construction of plasmid pT-mdtG |
| mdtG-UR | gcggattgctTTTTTCGCCTTTCATACTTGCAAAAG |  |
| mdtG-DF | aggcgaaaaaAGCAATCCGCTGTTGGTGC | Used for the construction of plasmid pT-mdtG |
| mdtG-DR | ggagctgcacatgaactcgagTTTTCGTCCCGTTGTTTGCC |  |
| mdtG-SPF | accaacagtgtctgtacggcgttttagagctagaaatagcaag | Used for the construction of plasmid pT-mdtG |
| mdtG-SPR | gccgtacagacactgttggttagtattatacctaggactgagc |  |
| mdtG-VF | AGTGGCTGTGGCAGCATTAT | Used for the verification and sequence of MGΔmdtG strain |
| mdtG-VR | ATGTGGTTACACCGTCGCTT |  |
| focB-UF | agagtcgacctgcagaagcttACCGTCCGGAAGATATCCCT | Used for the construction of plasmid pT-focB |
| focB-UR | gggctgattttGCATACTCCTGCAAAAGCAGATT |  |
| focB-DF | ggagtatgcAAAATCAGCCCGGCGAAAC | Used for the construction of plasmid pT-focB |
| focB-DR | ggagctgcacatgaactcgagGATGATCGTGGTGGGGATCG |  |
| focB-SPF | gacggtgatggcaaaaagtcgttttagagctagaaatagcaag | Used for the construction of plasmid pT-focB |
| focB-SPR | gactttttgccatcaccgtctagtattatacctaggactgagc |  |
| focB-VF | CCGCCAGTCTTCTGGAAAGT | Used for the verification and sequence of MGΔfocB strain |
| focB-VR | CTGGACGGCAACCTGTTAGT |  |
| pB-marB-F | tttttttgggctagcgaattcTATGGCTTCGAGTCGCAACA | Used for the construction of plasmid pB-marB |
| pB-marB-R | tccgccaaaacagccaagcttACGCTTAACGGGTCTGCAAT |  |
| pB-alx-F | tttttttgggctagcgaattcGGCAACCCGAAAGGAATACG | Used for the construction of plasmid pB-alx |
| pB-alx-R | tccgccaaaacagccaagcttCCAGCATCGGTGCGAAAAAT |  |
| pB-arsB-F | tttttttgggctagcgaattcCCTGGCGATGTGAACAGGAA | Used for the construction of plasmid pB-arsB |
| pB-arsB-R | tccgccaaaacagccaagcttAATAGTCGGTTCTGTGCCGC |  |
| pB-rcnA-F | tttttttgggctagcgaattcCACGCGCTTTCAGTTTCTGT | Used for the construction of plasmid pB-rcnA |
| pB-rcnA-R | tccgccaaaacagccaagcttCCTTAGCGAGCAGAGAGTCAG |  |
| pB-mdtL-F | tttttttgggctagcgaattcTGATTTTGTGCTGGTTCGGC | Used for the construction of plasmid pB-mdtL |
| pB-mdtL-R | tccgccaaaacagccaagcttTTGCCAGCGACTTACTCACC |  |
| pB-mdtG-F | tttttttgggctagcgaattcATGTGGTTACACCGTCGCTT | Used for the construction of plasmid pB-mdtG |
| pB-mdtG-R | tccgccaaaacagccaagcttGCAGCGTCAATGGCTTCTTC |  |
| pB-focB-F | tttttttgggctagcgaattcGAACGTCAGCGCATTGTTCA | Used for the construction of plasmid pB-focB |
| pB-focB-R | tccgccaaaacagccaagcttCCTGCATCCGCTGGTGATTA |  |
| pB-VF | GCCTCAATCGGCGTTAAACC | Used for the verification and sequence of pBAD18 series plasmids |
| pB-VR | ACGGCGTTTCACTTCTGAGT |  |
| ompA-QF | GACCCTGGTTGTAAGCGTCA | Used for qPCR detection of ompA gene |
| ompA-QR | GCACCGGAAGTACAGACCAA |  |
| gapA-QF | GATGGCCCGTCTCACAAAGA | Used for qPCR detection of gapA gene |
| gapA-QR | CAGACGAACGGTCAGGTCAA |  |
| mgtA-QF | TTATCGCGCTAATGGTCGCT | Used for qPCR detection of mgtA gene |
| mgtA-QR | ATAATATCGCCGGGCACCAG |  |
| corA-QF | ATCTGAACTTGGCCAGAGCC | Used for qPCR detection of corA gene |
| corA-QR | ACGCTCACGCAGAGTAAACA |  |
| kefB-QF | AGCGTTGTGAAGAAGCGGTA | Used for qPCR detection of kefB gene |
| kefB-QR | TCGGCATGTCACTCAACCTC |  |
| hcr-QF | TAGTTACGCCACTCATCGGC | Used for qPCR detection of hcr gene |
| hcr-QR | CGCGGTGATTATCTCTGGCT |  |
| sodC-QF | TCGGTAGCTTTGCCGTCATT | Used for qPCR detection of sodC gene |
| sodC-QR | TTCGCCCGATCTGAAAGCAT |  |
| sodB-QF | AAGGTACCGCGTTTGAAGGT | Used for qPCR detection of sodB gene |
| sodB-QR | TGCGCTTTGAAATCGGCAAA |  |
| sodA-QF | AAACCGTACTGCGCAACAAC | Used for qPCR detection of sodA gene |
| sodA-QR | GGAAGCTGCCGCTTTTTCAA |  |
| PmrD-QF | TCGGGGCTATAACTGCTTGC | Used for qPCR detection of PmrD gene |
| PmrD-QR | GGCGGCGCGATAAAAATGAT |  |
| PagP-QF | AAAGGAAACTGGCATGGCCT | Used for qPCR detection of PagP gene |
| PagP-QR | CAATGGCAGTAGAACCGGGA |  |
| phoP-QF | AATGGTATGGCTTTCCCGCA | Used for qPCR detection of phoP gene |
| phoP-QR | TCACAGGTCATTTCGCTCCC |  |
| phoQ-QF | CTTGCCCAGGGCGTAAAGTA | Used for qPCR detection of phoQ gene |
| phoQ-QR | TGGGCAACGTGCTGGATAAT |  |
| rcnA-QF | TCTTCAGCAAGGAAACGCCT | Used for qPCR detection of rcnA gene |
| rcnA-QR | CCCGCCAAAGGCAATTAACC |  |
| marR-QF | GGATATTACCGCGGCACAGT | Used for qPCR detection of marR gene |
| marR-QR | CAACCTTTCCACCCAGCCTT |  |
| marB-QF | AGCAGCTGCGCTTATTCTCT | Used for qPCR detection of marB gene |
| marB-QR | CGAGCGCATCCGACTTATCA |  |
| alx-QF | TATCTTCGGTGCCTTCCTGC | Used for qPCR detection of alx gene |
| alx-QR | TGTGGCGTACAGCAATCCAT |  |
| marA-QF | CTGGAATCGCCACTGTCACT | Used for qPCR detection of marA gene |
| marA-QR | CCTTCAGCTTTTGCGCGATT |  |
| arsB-QF | ATGCTGCTGGCTTTAGGGTT | Used for qPCR detection of arsB gene |
| arsB-QR | ACCATCACCGAGGCGTATTC |  |
| entS-QF | AGGGCGGATTAACGGTTTGT | Used for qPCR detection of entS gene |
| entS-QR | CTCCACCAGCACCAGCAATA |  |
| acrB-QF | TCGAAGCGATCATCCTCGTG | Used for qPCR detection of acrB gene |
| acrB-QR | TTCTTCCGCCATAACACGCT |  |
| aaeA-QF | TTACTCGAGGATCAACGGCG | Used for qPCR detection of aaeA gene |
| aaeA-QR | GCAAGACGCACCCATTCAAG |  |
| emry-QF | TGGAGCGATCGTCCTTATGC | Used for qPCR detection of emry gene |
| emry-QR | CGCCAGTAATAGCAAACCGC |  |
| emrB-QF | CTTCGGCGCTATTGTTCTGC | Used for qPCR detection of emrB gene |
| emrB-QR | GACGCGCCAAAATCCATACC |  |
| focB-QF | CCGCAGTAAAGTGTCGGTCT | Used for qPCR detection of focB gene |
| focB-QR | CCGACTTTTTGCCATCACCG |  |
| mdtG-QF | TACTTGGCGGATTTGTCCCC | Used for qPCR detection of mdtG gene |
| mdtG-QR | GAATACCGGACGTAAGCCGT |  |
| bcr-QF | CCGCATTGGCGCGTTAAATA | Used for qPCR detection of bcr gene |
| bcr-QR | GCCATCGCATTGGATGACAC |  |
| ybhF-QF | CCGTGAATTTTGGCTGCACA | Used for qPCR detection of ybhF gene |
| ybhF-QR | TCGTTAGCCGACTGTGCTTT |  |
| emrE-QF | ACACGGTTATGGCCATCTGT | Used for qPCR detection of emrE gene |
| emrE-QR | TGCCTATAATGGCTGGCAGG |  |
| mdtL-QF | TTAAAAGAAACGCGCCCAGC | Used for qPCR detection of mdtL gene |
| mdtL-QR | AGCAATACCGGTGACGTGTT |  |
| mntP-QF | GCCACCCTCCATAAACCGAA | Used for qPCR detection of mntP gene |
| mntP-QR | CCAGTAGCCAGAAACCGTGT |  |
| focA-QF | GGGGTCAGTTGGCGAAAAAC | Used for qPCR detection of focA gene |
| focA-QR | CCAAGACAGACGGCCTCAAT |  |
| mdtJ-QF | ATGCGCTGTGGGAAGGTATC | Used for qPCR detection of mdtJ gene |
| mdtJ-QR | CAGGTTTACGCGCTTTACGG |  |
| eGFP-pA-PrrnB-F | TTAGTACAGCTCGTCCATGCC | Used for the construction of plasmid pA-P*_rrnB_*-*eGFP* |
| eGFP-pA-PrrnB-R | tgcgccaccaCCTCTAGAAATAATTTTGTTTAACTTTAAGAAGGAGATATACCAAGCTTATGGTGAGCAAGGGCGAG |  |
| pA-PrrnB-F | GCATGGACGAGCTGTACTAAGGATCCTATTCAGGCGTAGCACC | Used for the construction of plasmid pA-P*_rrnB_*-*eGFP* |
| pA-PrrnB-R | TTTCTAGAGGtggtggcgcattatagggagttattccggcctgacaagaggaCTCGAGCCTTGAGAGCCTTCAACCCA |  |
| mgtA-FLAG-UF | agagtcgacctgcagaagcttggatctggggcgctttatgat | Used for the construction of plasmid pT-mgtA-FLAG |
| mgtA-FLAG-UR | TGGTGGCTGCCGCCGCCGCCGCTGCCGCCGCCGCCttgccagccgtaacgacg |  |
| mgtA-FLAG-DF | GGCGGCGGCGGCAGCgactacaaggatgacgatgacaagtaaagaataaactgggcacgatagccc | Used for the construction of plasmid pT-mgtA-FLAG |
| mgtA-FLAG-DR | ggagctgcacatgaactcgagTGAAAGTGGGCGACATCGTT |  |
| mgtA-FLAG-SPF | gctggcaataaagaataaacgttttagagctagaaatagcaag | Used for the construction of plasmid pT-mgtA-FLAG |
| mgtA-FLAG-SPR | gtttattctttattgccagctagtattatacctaggactgagc |  |
| mgtA-FLAG-VF | TTTTCACCGCTGGCCAGTTA | Used for the verification and sequence of strain MG-mgtA-FLAG |
| mgtA-FLAG-VR | ACGTCAGTCGCTGGATAACG |  |
| corA-FLAG-UF | agagtcgacctgcagaagcttCCTACGAGTTGCTGCTGGAT | Used for the construction of plasmid pT-corA-FLAG |
| corA-FLAG-UR | gtcGCTGCCGCCGCCGCCGCTGCCGCCGCCGCCcaaccagttcttccgcttaaagtacag |  |
| corA-FLAG-DF | GCGGCGGCGGCGGCAGCgactacaaggatgacgatgacaagtaaaaaacgagagcggtggct | Used for the construction of plasmid pT-corA-FLAG |
| corA-FLAG-DR | ggagctgcacatgaactcgagGCCTGTTCAGGCAATGGTTC |  |
| corA-FLAG-SPF | ctggttgtaaaaaacgagaggttttagagctagaaatagcaag | Used for the construction of plasmid pT-corA-FLAG |
| corA-FLAG-SPR | ctctcgttttttacaaccagtagtattatacctaggactgagc |  |
| corA-FLAG-VF | CCTGGCACCGTATCTGTACT | Used for the verification and sequence of strain MG-corA-FLAG |
| corA-FLAG-VR | CAGCGACTCCGTGATAGTGG |  |
| mgtS-FLAG-UF | agagtcgacctgcagaagcttAACAGCCTGCTATTGTGGGG | Used for the construction of plasmid pT-mgtS-FLAG |
| mgtS-FLAG-UR | gtcatcgtcatccttgtagtcaccaccgtcatcccatttgtggctgaaat |  |
| mgtS-FLAG-DF | ggtgactacaaggatgacgatgacaagtaatgaacggagataatccctcacct | Used for the construction of plasmid pT-mgtS-FLAG |
| mgtS-FLAG-DR | ggagctgcacatgaactcgagACATTACCGTGACAGCAGGT |  |
| mgtS-FLAG-SPF | caaatgggatgactaatgaagttttagagctagaaatagcaag | Used for the construction of plasmid pT-mgtS-FLAG |
| mgtS-FLAG-SPR | ttcattagtcatcccatttgtagtattatacctaggactgagc |  |
| mgtS-FLAG-VF | GCCGGGCGAGTATATGTTGA | Used for the verification and sequence of strain MG-mgtS-FLAG |
| mgtS-FLAG-VR | TGGTCATTGGAAGAGCGGAC |  |

The uppercase represented the sequences of paired sites and the lowercase represented the sequences for aligning.

**Legends for Movies S1**

To visualize the content fluctuation of cellular Mg^2+^, this video shows time-lapse fluorescence microscopy of E. coli cells cultured with (right) or without (left) 0.5 μg/ml tellurite. The cells were strained with a Mg^2+^-specific dye (Mago-fluo-4, AM), and spotted onto an agarose pad. Images were captured at 30-second intervals over a 20-minute period following the administration of tellurite. The results demonstrated that the treated cells exhibited a more rapid leakage of intracellular magnesium. Video is representative of three independent replicates.

**Reference**

1. Fontoura I, Veriato TS, Raniero LJ, Castilho ML. Analysis of capped silver nanoparticles combined with imipenem against different susceptibility profiles of *Klebsiella pneumoniae*. Antibiotics. 2023; **12**: 535.

2. Ansari MA, Khan HM, Khan AA, Cameotra SS, Pal R. Antibiofilm efficacy of silver nanoparticles against biofilm of extended spectrum β-lactamase isolates of *Escherichia coli* and *Klebsiella pneumoniae*. Applied Nanoscience. 2013; **4**: 859‒868.

3. Vaidya M, McBain AJ, Banks CE, Whitehead KA. Single and combined antimicrobial efficacies for nine metal ion solutions against *Klebsiella pneumoniae*, *Acinetobacter baumannii* and *Enterococcus faecium*. Int Biodeter Biodegr. 2019; **141**: 39‒43.

4. Ning C, Wang X, Li L, Zhu Y, Li M, Yu P, et al. Concentration ranges of antibacterial cations for showing the highest antibacterial efficacy but the least cytotoxicity against mammalian cells: implications for a new antibacterial mechanism. Chem Res Toxicol. 2015; **28**: 1815‒1822.

5. Matarlo JS, Evans CE, Sharma I, Lavaud LJ, Ngo SC, Shek R, et al. Mechanism of MenE inhibition by acyl-adenylate analogues and discovery of novel antibacterial agents. Biochemistry. 2015; **54**: 6514‒6524.

6. Edziri H, Mastouri M, Mahjoub MA, Mighri Z, Mahjoub A, Verschaeve L. Antibacterial, antifungal and cytotoxic activities of two flavonoids from *Retama raetam* flowers. Molecules. 2012; **17**: 7284‒7293.

7. Guzman LM, Belin D, Carson MJ, Beckwith J. Tight regulation, modulation, and high-level expression by vectors containing the arabinose PBAD promoter. J Bacteriol. 1995; **177**: 4121‒4130.

8. Jiang Y, Chen B, Duan C, Sun B, Yang J, Yang S. Multigene editing in the *Escherichia coli* genome via the CRISPR-Cas9 system. Appl Environ Microbiol. 2015; **81**: 2506‒2514.
