# Supplementary material for: The antibacterial effect of tellurite is achieved through intracellular acidification and magnesium disruption
Source: mLife. 2025 Aug 24;4(4):423–36. doi: 10.1002/mlf2.70028 (PMC12395589; doi:10.1002/mlf2.70028)
Supplement: Supplementary file 5 — Fig‐S5. [file MLF2-4-423-s017.pdf]

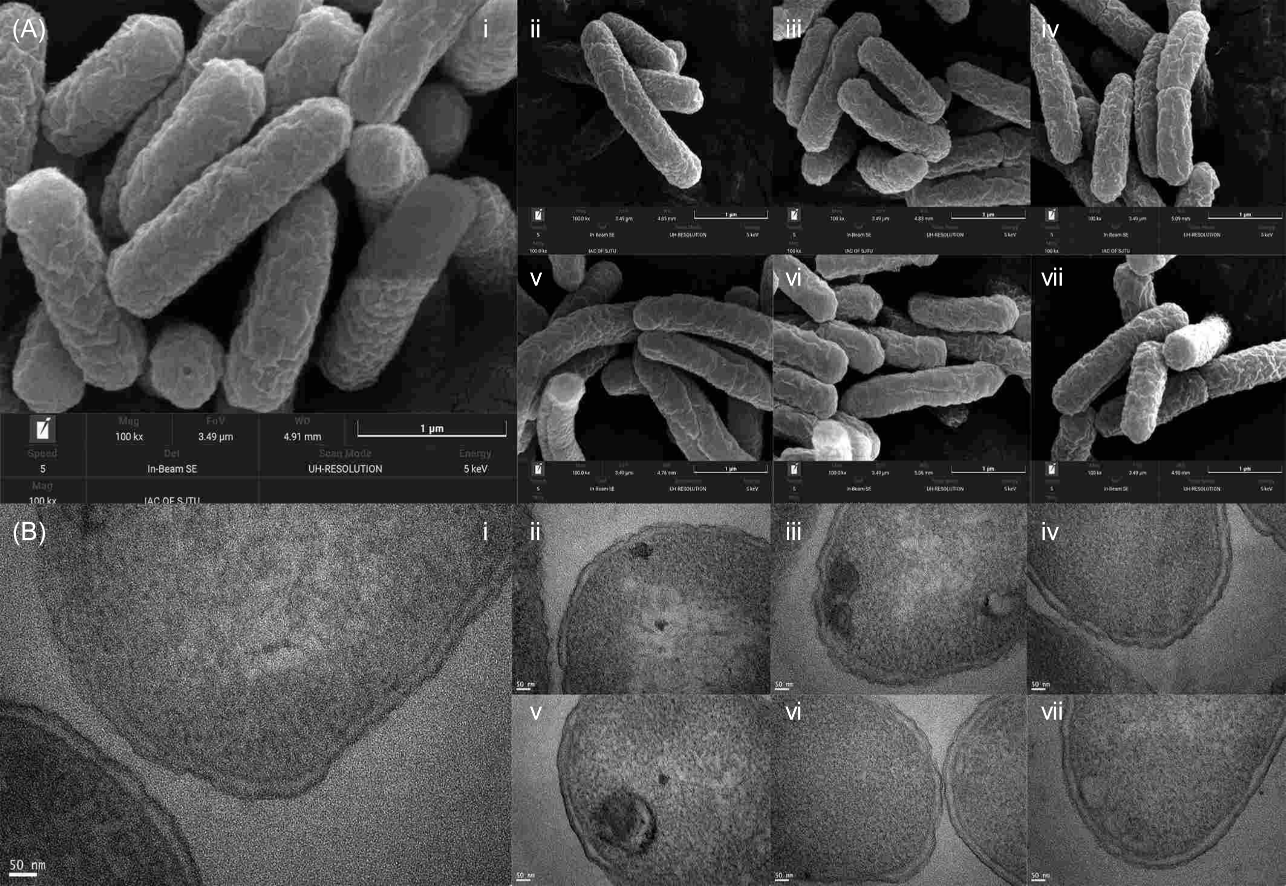
(A)
i
ii
iii
iv
v
vi
vii
Mag
100 kx
FoV
3.49 μm
WD
4.91 mm
1 μm
Speed
5
Det
In-Beam SE
Scan Mode
UH-RESOLUTION
Energy
5 keV
(B)
50 nm
